# Atrial cardiomyopathy: markers and outcomes

**DOI:** 10.1093/eurheartj/ehaf793

**Published:** 2025-10-15

**Authors:** Oliver B Vad, Nick van Vreeswijk, Ahmed S Yassin, Yuri Blaauw, Christian Paludan-Müller, Jørgen K Kanters, Claus Graff, Ulrich Schotten, Emelia J Benjamin, Jesper H Svendsen, Michiel Rienstra

**Affiliations:** Department of Cardiology, University of Groningen, University Medical Center Groningen, Groningen, The Netherlands; Department of Cardiology, Copenhagen University Hospital—Rigshospitalet, Copenhagen, Denmark; Department of Biomedical Sciences, University of Copenhagen, Copenhagen, Denmark; Department of Cardiology, University of Groningen, University Medical Center Groningen, Groningen, The Netherlands; Department of Cardiology, University of Groningen, University Medical Center Groningen, Groningen, The Netherlands; Department of Cardiology, University of Groningen, University Medical Center Groningen, Groningen, The Netherlands; Department of Cardiology, Copenhagen University Hospital—Rigshospitalet, Copenhagen, Denmark; Department of Cardiothoracic Surgery, Copenhagen University Hospital—Rigshospitalet, Copenhagen, Denmark; Department of Biomedical Sciences, University of Copenhagen, Copenhagen, Denmark; Center of Biosignal Research, University of California, San Francisco, CA, USA; Department of Health Science and Technology, Aalborg University, Aalborg, Denmark; Department of Physiology, Cardiovascular Research Institute Maastricht, Maastricht, The Netherlands; Department of Medicine, Chobanian and Avedisian School of Medicine, Boston Medical Center, Boston University, Boston, MA, USA; Department of Epidemiology, Boston University School of Public Health, Boston, MA, USA; Department of Cardiology, Copenhagen University Hospital—Rigshospitalet, Copenhagen, Denmark; Department of Clinical Medicine, University of Copenhagen, Copenhagen, Denmark; Department of Cardiology, University of Groningen, University Medical Center Groningen, Groningen, The Netherlands

**Keywords:** Atrial cardiomyopathy, Atrial fibrillation, Heart failure, Stroke, UK Biobank

## Abstract

**Background and Aims:**

Atrial cardiomyopathy (AtCM) is increasingly recognized as an important substrate for atrial fibrillation (AF). This study aimed to examine potential markers and risk factors of AtCM, and associations with incident AF, heart failure (HF), and stroke.

**Methods:**

Individuals from the UK Biobank with cardiac magnetic resonance imaging and electrocardiographic information were included. Atrial cardiomyopathy markers included left atrial dilation, left atrial mechanical dysfunction, P-wave prolongation, and abnormal P-wave terminal force. Risk factors for AtCM were assessed using logistic regressions. Incident AF, HF, and stroke according to AtCM markers were assessed in multivariable Cox-regression and cumulative incidence models. AF risk according to AtCM markers, clinical and genetic risk factors was evaluated by integrating the HARMS_2_-AF score and a polygenic risk score for AF. We used net reclassification improvement (NRI) to evaluate reclassification of risk when considering AtCM markers.

**Results:**

Among 26 467 individuals, 4145 (15.7%) had ≥1 marker and 619 (2.3%) had ≥2 markers of AtCM. Age, coronary artery disease, and hypertension were consistently associated with AtCM. Having one AtCM marker conferred a hazard ratio (HR) for AF of 1.88 [95% confidence interval (CI): 1.54–2.31; *P* < .001], with higher rates observed in individuals with ≥2 markers (HR: 4.59; 95% CI: 3.52–5.99; *P* < .001). Addition of AtCM markers was associated with an NRI of 13.7% (95% CI: 9.2%–18.3%). Integration of clinical and genetic risk factors indicated an additive effect on AF rates. Having ≥2 markers associated with HF (HR: 3.08, 95% CI: 2.03–4.66, *P* < .001), and stroke (HR: 3.07, 95% CI: 1.78–5.28, *P* < .001).

**Conclusions:**

One in seven individuals had at least one marker of AtCM. Atrial cardiomyopathy markers were associated with AF, HF, and stroke, supporting AtCM as a common substrate for all three outcomes.

## Structured Graphical Abstract

**Structured Graphical Abstract:**
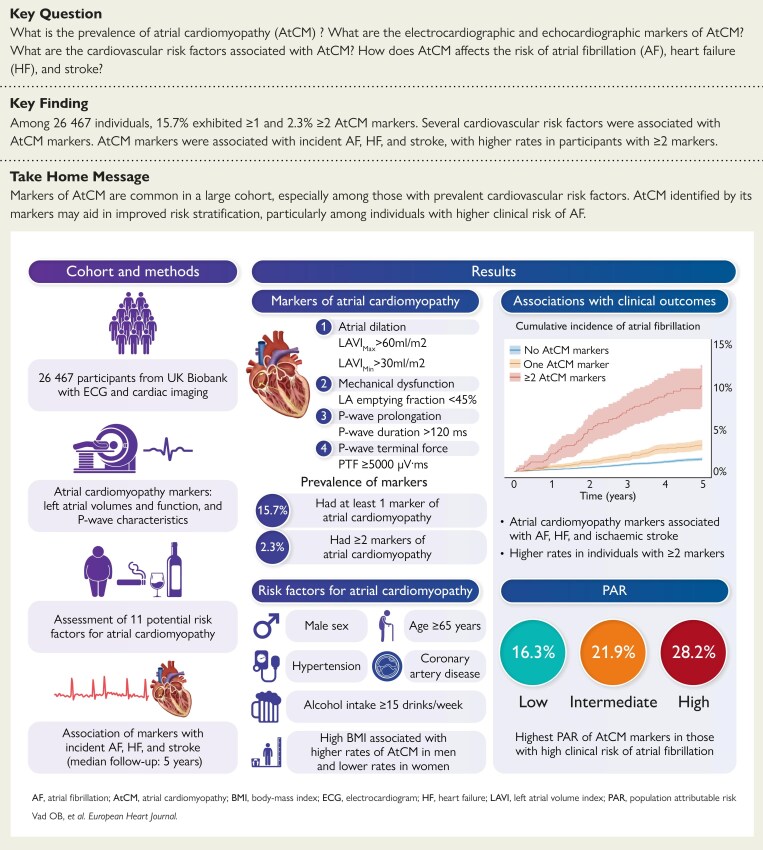
Structured graphical abstract showing the methods, study design, and main findings. A total of 26 467 individuals from the UK Biobank were examined, focusing on four markers of atrial cardiomyopathy. AF, atrial fibrillation; AtCM, atrial cardiomyopathy; BMI, body mass index; ECG, electrocardiogram; HF, heart failure; LAVI_Max_, maximum left atrial volume index; LAVI_Min_, minimum left atrial volume index; PAR, population attributable risk; PTF, P-wave terminal force.

**See the editorial comment for this article ‘Understanding atrial cardiomyopathy: a missing link in the prevention of atrial fibrillation, heart failure, and stroke?', by M.R. Schubert and M.F. Sinner, https://doi.org/10.1093/eurheartj/ehaf832.**

## Introduction

Atrial cardiomyopathy (AtCM) is a complex and heterogeneous condition characterized by structural, functional, or electrophysiological remodelling of the atrial myocardium, which predisposes to adverse clinical outcomes,^1^ such as atrial fibrillation (AF), stroke, and heart failure (HF).^2–4^ Despite its conceptual relevance, the diagnosis of AtCM remains challenging due to the lack of a consensus definition and standardized diagnostic markers.

Indirect measures, such as imaging-based left atrial (LA) volumes, atrial function, and electrocardiogram (ECG)-based prolonged P-wave duration have been proposed,^5–7^ but AtCM itself is in most patients asymptomatic, allowing it to progress unnoticed. In practice, AtCM is investigated after the development of AF, stroke, or HF, and it is unknown whether early detection of AtCM can provide an opportunity to slow progression of AtCM and reduce AtCM-related AF, stroke, or HF.

Atrial fibrillation risk factors such as hypertension, diabetes, obesity, smoking, and alcohol use are well-established contributors to AF, stroke, and HF initiation and perpetuation.^8–11^ These comorbidities and risk factors may also accelerate the development and progression of AtCM, affecting the substrate of AF, stroke, and HF.^12^

Examining how potential risk factors influence AtCM in a cohort without concurrent AF or HF could help bridge knowledge gaps on the development of AtCM. An improved characterization and understanding of AtCM might aid in identification of individuals at risk of adverse clinical outcomes.^13^

In this study, we integrated clinical, cardiac imaging, ECGs, and genetic information from a large biobank, to ascertain markers for AtCM and associations with both comorbidities and risk factors, and with incident AF, stroke, and HF. We aimed to: (i) examine referent ranges for cardiac magnetic resonance imaging (cMRI)-based LA measures and assess cut-off values for AtCM markers, (ii) evaluate the prevalence of these markers and identify potential risk factors for AtCM, (iii) examine associations with AF, HF, and stroke, and (iv) study the integration of AtCM markers with clinical and genetic risk scores.

## Methods

### Study cohort

The study was conducted using data from the UK Biobank, a large population-based biobank of more than 500 000 individuals. The composition of the biobank, including genotyping, has previously been described in detail.^14^ The UK Biobank received ethical approval from the North West Multi-centre Research Ethics Committee in the United Kingdom (#11/NW/0382), and all participants gave written, informed consent.

Participants of European ancestry with complete ECG and cMRI data were included. We used a complete-case approach for handling missing data, and excluded individuals with missing data on sex, body mass index (BMI), self-reported alcohol intake, or smoking status. Individuals with disagreement between reported and genetically determined sex were excluded. Individuals with a history of AF, stroke, or HF, prior to date of imaging and ECG were excluded, as were individuals not in sinus rhythm at the time of ECG. Diagnoses were defined using ICD-10 codes from primary care, hospital records, and death registries. All phenotype definitions and corresponding UK Biobank data fields are summarized in Supplementary data online, *Table S1*. A flowchart of cohort participant selection for the study cohort is provided in Supplementary data online, *Figure S1*.

### Markers of atrial cardiomyopathy

We selected markers representing electrical and structural remodelling of the left atrium, including LA dilation, reduced LA emptying fraction, prolonged P-wave duration, and abnormal P-wave terminal force (PTF). Left atrial measures were derived from cMRI, as described by Bai *et al.*,^15^ and volumes were indexed using body surface area (BSA) at time of imaging to allow comparisons between individuals with different body sizes.^16,17^ P-wave duration was extracted from the automated readings of 12-lead at-rest ECGs, taken at the same visit as first imaging visit. P-wave prolongation was defined as P-wave >120 ms based on previous literature.^18^ To calculate PTF, we extracted P-wave amplitude and duration of the second negative part of the P-wave in lead V1. PTF was calculated as the duration * amplitude of the negative part of the P-wave in lead V1. If a negative P-wave component did not exist, PTF was set to 0. PTF ≥ 5000 µV ms was considered abnormal, in accordance with previous literature.^12^

For cardiac magnetic resonance-derived measures, we set thresholds based on previously published literature on other populations^19^ and guidelines.^20^ To improve interpretability and provide clinically meaningful cutoffs, we applied rounding of the cutoffs for LA volumes and LA_EF_. Left atrial dilation was defined as indexed LA volumes above upper reference range (either LAVI_Max_ > 60 mL/m^2^ or LAVI_Min_ > 30 mL/m^2^), and mechanical dysfunction as LA emptying fraction (LA_EF_) < 45%. To evaluate the applicability of these thresholds in this dataset, we estimated reference ranges for each measure by calculating the mean ± 1.96 standard deviation (SD).

### Risk factors for atrial fibrillation and influence on atrial cardiomyopathy

We evaluated established risk factors for AF, including diagnosed coronary artery disease (CAD), diabetes, hypertension, chronic obstructive pulmonary disease (COPD), sleep apnea, and chronic kidney disease (CKD), sex, age, obesity (defined as BMI ≥30 kg/m^2^), and self-reported smoking status, and alcohol intake at enrolment. Age was stratified by age at or above, or below 65 years at time of imaging. Smoking status was stratified by never (reference group), previous, or current smoking, and alcohol intake was stratified by <7 drinks per week (reference group), 7–14 drinks per week, and ≥15 drinks per week. Risk factors were also evaluated in sex-stratified analyses, and for each AtCM marker separately.

### Clinical and genetic risk scores

Clinical risk was ascertained using the HARMS_2_-AF score, which has previously been evaluated in the UK Biobank and the Framingham cohort.^21^ This clinical risk score includes the components sex, age, obesity, hypertension, sleep apnea, smoking, and alcohol intake. Clinical risk was stratified by HARMS_2_-AF score ≥10 (high risk), HARMS_2_-AF score between 5 and 9 (intermediate risk), and HARMS_2_-AF score <5 (low risk).

Genetic risk of AF was defined using a polygenic risk score (PRS) for AF. We used weights from a previously published PRS based on an AF genome-wide association study that did not overlap with the UK Biobank.^22,23^ PRS weights were obtained from the Cardiovascular Disease Knowledge Portal (https://cvd.hugeamp.org), and were calculated using the continuous shrinkage (PRS-cs) algorithm, which leverages a Bayesian regression framework i.e. robust to varying genetic architectures.^24^ The PRS has previously been evaluated in the Stroke Genetics Cohort^23^ and the UK Biobank.^25^

Two PRSs were used to reflect genetic risk of HF and ischemic stroke, respectively. For HF, we obtained weights from a previously published PRS through the PGS catalog^26^ (PRS identifier: PGS001790). The HF score was calculated using the PRS-cs algorithm, and a detailed methodology has been described by Wang and colleagues.^27^ For ischemic stroke, we constructed a PRS based on publicly available genetic summary statistics for ischemic stroke from the MEGASTROKE consortium.^28^ PRS weights were calculated with the PRS-cs algorithm,^24^ using the European ancestry panel from the 1000 genomes project as a reference.^29^

Individual PRSs for all three outcomes were calculated for each participant in the cohort using PLINK,^30^ based on the number of risk alleles and posterior effect sizes of genetic variants. The PRSs were scaled to a mean of 0 and a SD of 1. Genetic risk was stratified by PRS ≥75th percentile (high risk), PRS between 25th and 74th percentile (intermediate risk), and PRS <25th percentile (low risk).

### Risk reclassification according to atrial cardiomyopathy markers

To assess the applicability of the four AtCM markers in risk stratification, we constructed net reclassification improvement (NRI) indexes, using the *nricens* package in R.^31^ We used three risk categories for 5-year risk of AF: < 5% risk, 5%–15% risk, and >15% risk. Event NRI (NRI^+^) was calculated by number of cases that were correctly reclassified into a higher risk category vs number of cases that were classified into a lower risk category. Non-event NRI (NRI^−^) was calculated by the proportion of non-cases that were reclassified into a higher risk category vs non-cases that were down-classified. Overall NRI was calculated as NRI = NRI^+^ + NRI^−^. Confidence intervals (CI) were calculated by bootstrapping.

For AF as an outcome, we compared a baseline model adjusted for sex, age, imaging centre, BMI, and history of CAD, hypertension, and diabetes, with a model with further adjustment for number of AtCM markers (none, one, or ≥2 AtCM markers). Reclassification in comparison with clinical and genetic risk scores was estimated by comparing models adjusted for clinical risk score categories and genetic risk score categories, respectively, as defined above, and models with further adjustment for the presence of none or ≥1 AtCM markers.

### Model calibration and discrimination

For incident AF, HF, and stroke, model discrimination was assessed using Harrel’s *C*-statistic. To assess the incremental value of AtCM markers, the models including AtCM markers were compared with a reference model adjusted only for sex, age, and BMI, and a model with further adjustment for hypertension, CAD, and diabetes. Calibration plots were used to determine potential over- or underestimation of risk by the models. The cohort was grouped into six risk bins, stratified at 1%, 2.5%, 5%, 7.5%, and 10% predicted risk for AF, and at 1%, 2%, 3%, 4%, and 5% predicted risk for HF, and stroke. Calibration was estimated with the rms package at 5 years of follow-up, using bootstrapping with 200 replicates.

### Statistical analyses

We described the baseline participant characteristics by number (%), mean ± SD, or median (Q1, Q3). We calculated the odds ratio (OR) for having ≥1 AtCM marker using logistic regression models. All risk factors were assessed separately in univariable models and in a multivariable model containing all risk factors as components. We tested for interaction with sex by including it as an interaction term and evaluated associations between risk factors and AtCM in multivariable sex-stratified analyses. As 11 different risk factors were evaluated, we adjusted for multiple testing with a Bonferroni correction, considering *P* < .0045 (.05/11) as statistically significant.

Hazard ratios (HR) for incident AF were estimated for each marker separately, and according to number of AtCM markers using multivariable Cox regressions. Date of first imaging visit was considered the index date, and participants were followed until first incident AF, death, or end of follow-up (1 January 2023), whichever came first. Median follow-up time represented time between imaging/ECG and first event. Models were adjusted for sex, age, imaging centre, BMI, and history of CAD, hypertension, and diabetes. Crude cumulative incidences for AF were calculated using the Aalen–Johansen estimator, considering all-cause mortality as a competing risk. For outcomes, we applied a Bonferroni correction and considered *P* < .016 (.05/3 outcomes) as statistically significant. Associations between AtCM markers and the outcomes were also evaluated for each marker separately, and pairwise combinations of the four different markers.

To account for a potential shared pathophysiology between AtCM, AF, and HF, we conducted sensitivity analyses censoring Cox regressions at first incident HF and calculated crude cumulative incidences considering HF as a competing risk. As secondary outcomes, we assessed incident stroke and HF during follow-up, in Cox models and cumulative incidence models as described above. We conducted sensitivity analyses considering AF as a competing risk. To account for potential undiagnosed left ventricular dysfunction, we conducted sensitivity analyses in a subset of the cohort, excluding individuals with a left ventricular ejection fraction (LVEF) < 50%.

Incident AF by the presence of ≥1 AtCM marker was also assessed in groups stratified by low, intermediate, and high clinical and genetic risk scores, respectively, using Cox-regression models and Aalen–Johansen cumulative incidence models. Hazard ratios stratified by clinical risk were assessed in univariable models, while HRs stratified by genetic risk were assessed in models with adjustment for sex, age at imaging, and six first genotype principal components (PCs). As a supplemental analysis, we also examined the cohort with further stratification for either no AtCM markers, 1 AtCM marker, or ≥2 AtCM markers, according to clinical and genetic risk. We examined the aggregate effect of both clinical and genetic risk, combined with AtCM markers, in models stratified by both clinical and genetic risk score categories and presence of ≥1 AtCM.

We further examined the combined role of genetic risk and AtCM markers for the outcomes HF and stroke by stratifying the cohort by genetic risk categories (as described above) and the presence of ≥1 AtCM. Models were adjusted sex, age at imaging, and six first genotype PCs.

We assessed the population attributable risk (PAR) of having an AtCM marker, stratified by clinical and genetic risk groups. PAR was calculated using the formula PAR = *P*_e_ × (HR − 1)/(1 + *P*_e_ × [HR − 1]) as previously described.^32^  *P*_e_ denotes the prevalence of the exposure (i.e. AtCM) and HR denotes the adjusted HR for AF.

All statistical analyses were conducted in R version 4.2.0,^31^ using the packages *survival* and *prodlim*. The proportional hazards assumption was evaluated using scaled Schoenfeld residuals.

### Combined risk model for atrial fibrillation

As an exploratory analysis, we constructed a weighted risk model for AF, combining clinical, genetic, imaging-based, and ECG-based risk markers. Clinical risk factors were stratified by HARMS2-AF score in one-point increments. Genetic risk was stratified by PRS for AF (PRS < 75th percentile, PRS between 75th and 94th percentile, or PRS ≥ 95th percentile). Markers of AtCM were divided into markers of electrical remodelling (either prolonged P-wave or abnormal PTF) and markers of structural remodelling (either LA dilation or LA mechanical dysfunction).

The cohort was split by a 70/30 proportion into training and testing datasets. An elastic net using a Cox proportional hazard model and a five-fold cross-validation with penalization of .9 was employed in the training set to combine all factors into a weighted risk model. To construct a weighted score, beta-coefficients were multiplied by an adjustment factor of 5.3, and rounded to nearest integer, corresponding to each 1-unit increment increase in HARMS2-AF score, yielding a one-point increase in the integrated risk score. The weighted model was then evaluated using univariable Cox-regressions, the testing dataset per unit increase. AF incidence per 100 person-years was evaluated for each 2-point increase in the integrated risk score. Internal validation with 200 bootstrap replicates was performed using the rms package in R to quantify potential optimism in the model.

## Results

We included 26 467 individuals with cMRI imaging of the left atrium and ECG data, without prior history of AF, HF, or stroke. Median age at imaging/ECG was 64.4 years (1st–3rd quartile: 58.1–69.8), and 13 914 (52.6%) of participants were women. Individuals’ characteristics at index date are summarized in *Table 1*.

**Table 1:** Baseline characteristics at imaging

Study cohort (*n* = 26 467)	
Sex	
Women, *n* (%)	13 914 (52.6)
Men, *n* (%)	12 553 (47.4)
Age, years, median (1st–3rd quartile)	64.4 (58.1–69.8)
Body mass index, kg/m^2^, mean ± SD	26.4 ± 4.3
Blood pressure	
Systolic, mmHg, mean ± SD	140 ± 20
Diastolic, mmHg, mean ± SD	79 ± 11
Cardiometabolic comorbidities	
Chronic kidney disease, *n* (%)	471 (1.8)
Chronic obstructive pulmonary disease, *n* (%)	286 (1.1)
Coronary artery disease, *n* (%)	1367 (5.2)
Diabetes, *n* (%)	1259 (4.8)
Hypertension, *n* (%)	7485 (28.3)
Obstructive sleep apnea, *n* (%)	215 (.8)
Smoking status	
Never smoking, *n* (%)	16 692 (63.1)
Previous smoking, *n* (%)	8860 (33.5)
Current smoking, *n* (%)	915 (3.5)
Alcohol intake	
<7 drinks per week, *n* (%)	11 358 (42.9)
7–14 drinks per week, *n* (%)	7297 (27.6)
≥15 drinks per week, *n* (%)	7812 (29.5)

Table of baseline characteristics at index date.

BP, blood pressure; SD, standard deviation.

**Figure 1:**
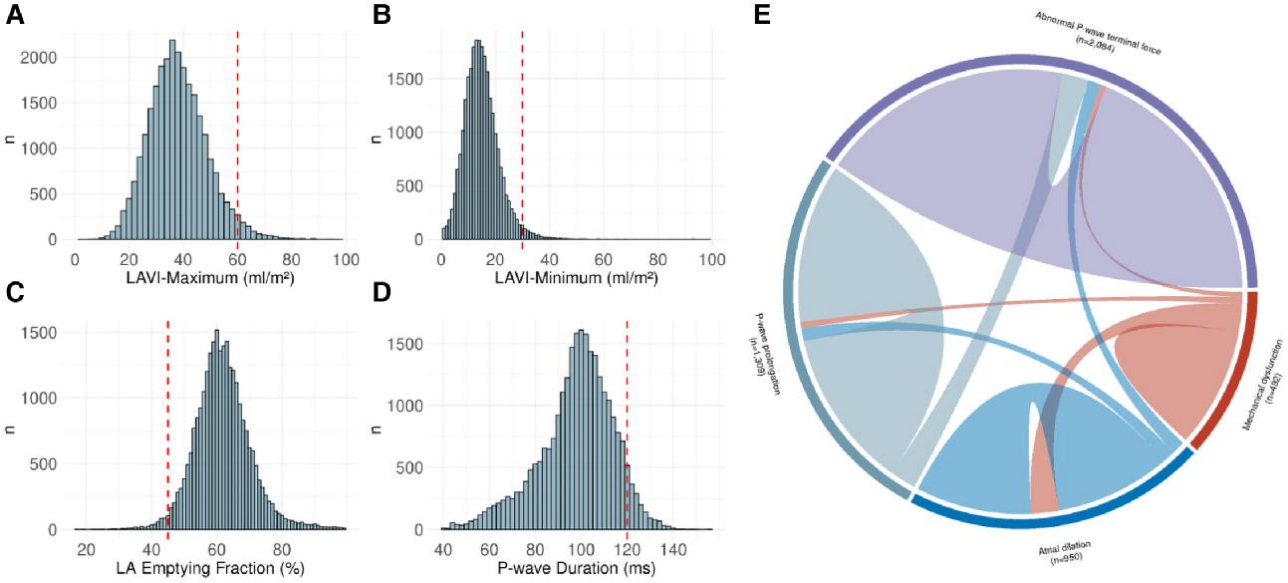
Cutoffs, prevalence, and co-occurrence of AtCM markers. LA, left atrial; VI, volume index. Panels *A–D* show histograms for LA volumes (*A* LAVI_Max_; *B* LAVI_Min_), function (*C* LA emptying fraction), and P-wave duration (*D*), with cutoff values marked with a red dotted line. Panel *E* shows a chord diagram of prevalence and co-occurrence of LA dilation, mechanical dysfunction (LA emptying fraction), and electrical dysfunction (P-wave duration)

In comparison to the thresholds defined for LA dilation (LAVI_Max_ > 60 mL/m^2^ or LAVI_Min_ > 30 mL/m^2^), the upper referent range for the cMRI-derived LA measures in the present study was observed to be 58.9 mL/m^2^ for LAVI_Max_, and 27.5 mL/m^2^ for iLA_Min_. The observed lower referent range was 45.0% for LA_EF_, similar to the threshold set at LA_EF_ < 45%. Histograms of all observations are shown in *Figure 1A–D*.

### Prevalence and inter-relations of atrial cardiomyopathy markers

We identified 4145 (15.7%) individuals with ≥1 AtCM marker, while 619 (2.3%) individuals had ≥2 markers of AtCM. The most frequent marker was abnormal PTF, observed in 2084 (7.9%) individuals, followed by prolonged P-wave (1309 [4.9%] individuals), LA dilation (950 [3.6%] individuals), and LA mechanical dysfunction (492 [1.9%] individuals). The co-occurrence of different markers is visualized in *Figure 1E*.

### Risk factors associated with atrial cardiomyopathy markers

**Figure 2:**
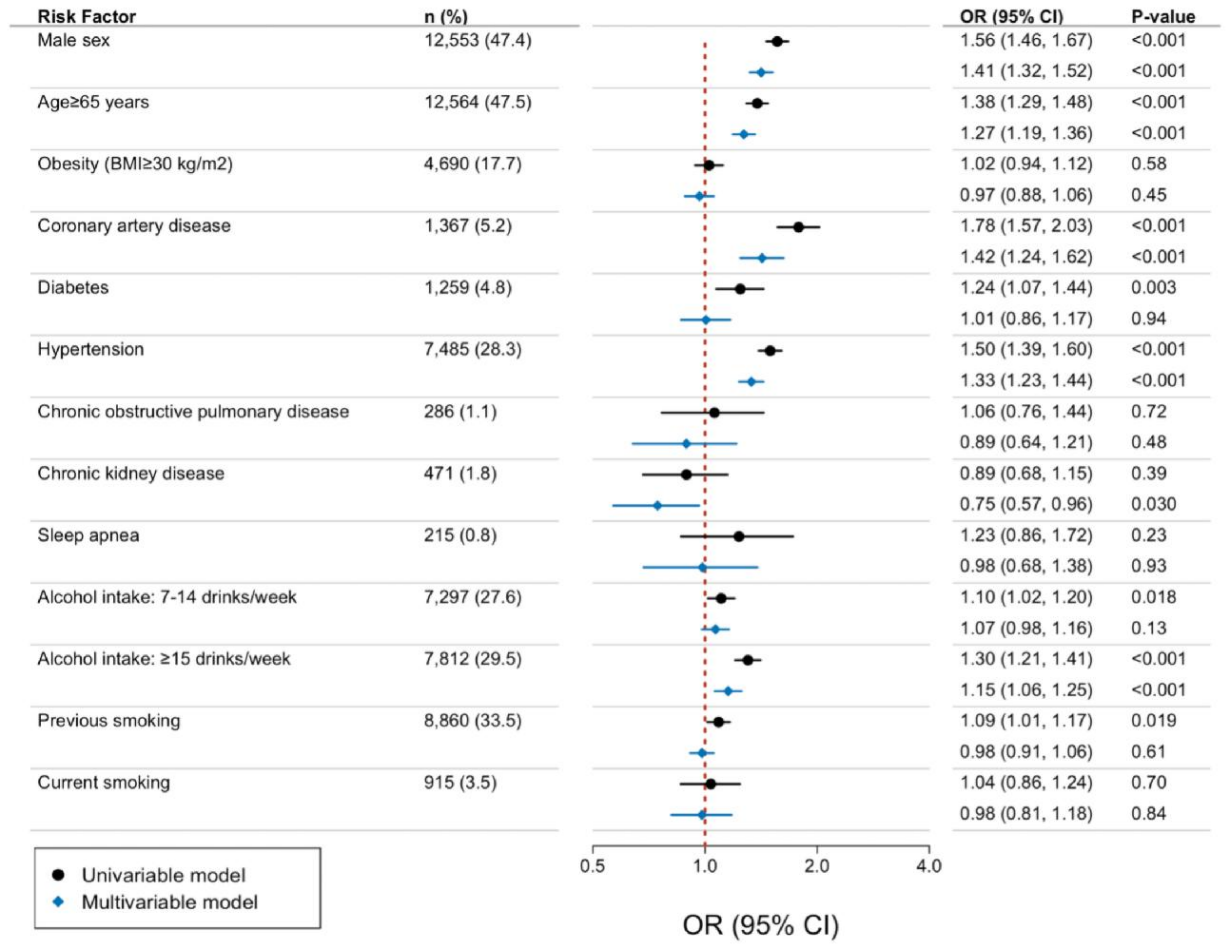
Risk factors associated with AtCM. Odds ratio and 95% confidence intervals for having ≥1 AtCM marker at time of imaging. Estimates provided for univariable and multivariable models. Red dotted line represents a reference odds ratio of 1.0. *P*-values <.0045 were considered statistically significant. BMI, body-mass index; CI, confidence intervals; OR, odds ratio

We examined associations between 11 established risk factors for AF, and AtCM markers. When evaluated in multivariable models, five risk factors were significantly associated with AtCM markers: male sex, age ≥65 years, history of CAD, history of hypertension, and alcohol intake ≥15 drinks/week. All risk factors, estimates, and *P*-values are summarized in *Figure 2*.

**Figure 3:**
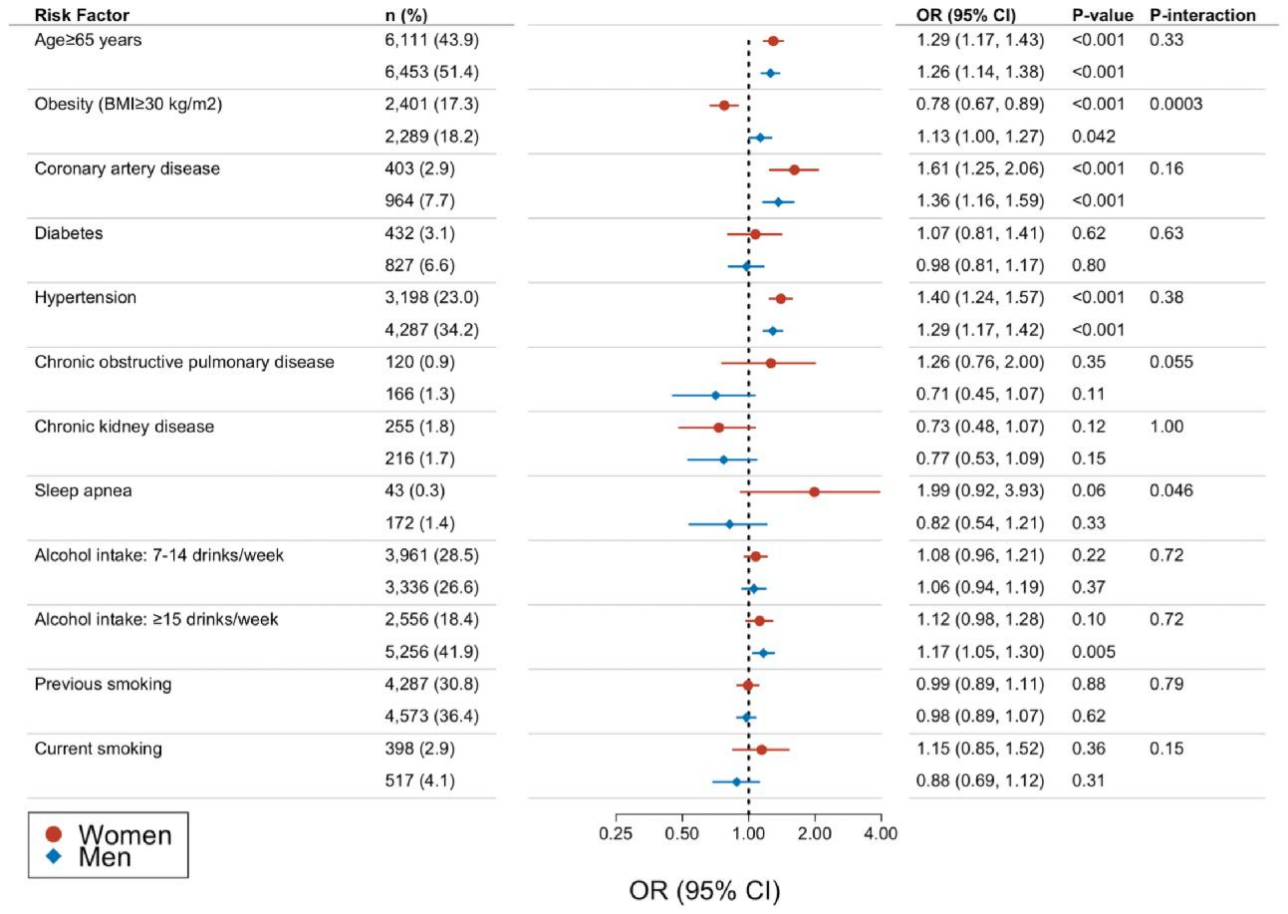
Risk factors associated with AtCM stratified by sex. Odds ratio and 95% confidence intervals for having ≥1 AtCM marker at time of imaging. Multivariable models, stratified by sex. Dotted line represents a reference odds ratio of 1.0. *P*-values <.0045 were considered statistically significant. *P*-values for interaction with sex are shown in right-most column. BMI, body-mass index; CI, confidence intervals; OR, odds ratio

In multivariable, sex-stratified analyses (*Figure 3*), three risk factors were consistently associated with the presence of an AtCM marker: age ≥65 years, history of CAD, and history of hypertension. We observed an interaction between sex and obesity (*P* < .001). Having a BMI ≥30 kg/m^2^ was associated with increased OR of AtCM in men (OR: 1.13, 95% CI: 1.00–1.27, *P* < .042) but a lower OR for AtCM in women (OR: .78, 95% CI: 0.67–0.89, *P* < .001). Hypertension was consistently associated with all four distinct markers when evaluated (see Supplementary data online, *Figure S2*). Sex-stratified associations between the AF risk factors showed an interaction between sex and alcohol intake ≥15 drinks/week for P-wave duration (*P* = .049), and between sex and obesity for LA dilation (*P* = .011). Sex-stratified results are summarized for each AtCM marker in Supplementary data online, *Figs S3*–*S6*.

### Incident atrial fibrillation according to atrial cardiomyopathy markers

During a median follow-up time of 4.9 years (1st–3rd quartile: 3.9–6.3 years), 560 (2.1%) individuals were diagnosed with first incident AF, and 411 (1.6%) individuals died before event or end of follow-up. We observed higher rates of AF with increasing number of AtCM markers. Having at least one marker of AtCM was associated with an increased HR of 2.35 (95% CI: 1.97–2.80, *P* < .001) for AF compared with individuals without a marker of AtCM.

**Figure 4:**
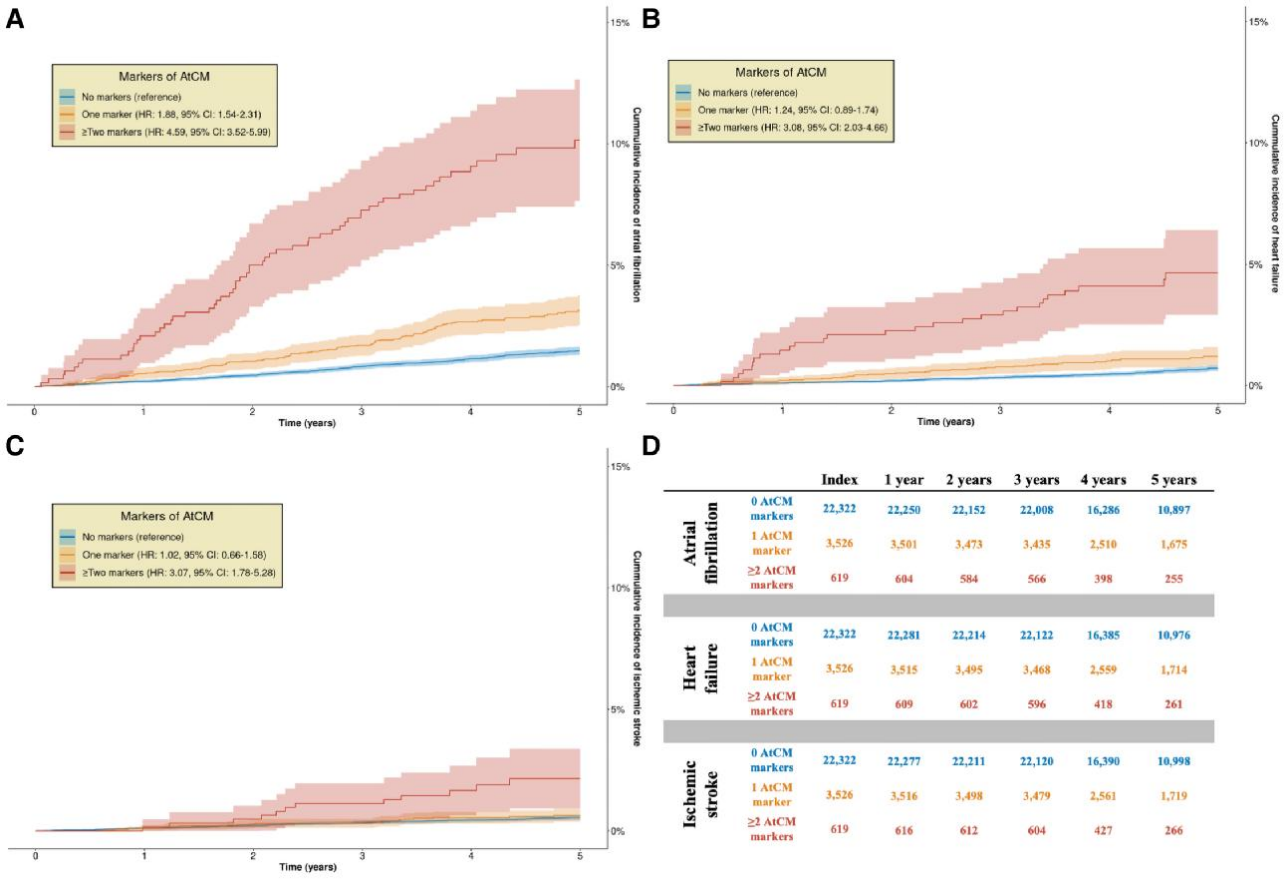
Rates of atrial fibrillation, heart failure, and ischemic stroke. Cumulative incidence and 95% confidence intervals stratified by number of AtCM markers. Panel *A* shows incidence of atrial fibrillation, panel *B* shows incidence of HF, and panel *C* shows incidence of ischemic stroke. Panel *D* shows number at risk at each time point for each respective outcome. *X*-axes show time since imaging/ECG in years. *Y*-axes show cumulative incidence of each outcome. AtCM, atrial cardiomyopathy, CI, confidence interval; HR, hazard ratio

Stratification by number of AtCM markers revealed a dose–response-like relationship. Having one AtCM marker was associated with a HR for AF of 1.88 (95% CI: 1.54–2.31; *P* < .001), while those with ≥2 markers had a HR of 4.59 (95% CI: 3.52–5.99, *P* < .001), compared with referents. At 5 years of follow-up after index date, those with no markers of AtCM had a 5-year AF incidence of 1.47% (95% CI: 1.30%–1.64%). In comparison, 5-year incidence was 3.15% (95% CI: 2.54%–3.77%) in individuals with 1 AtCM marker, and 10.16% (7.65%–12.66%) in individuals with ≥2 markers of AtCM. Results are visualized in *Figure 4A*. HRs for AF according to pairwise combinations of the different markers are summarized in Supplementary data online, *Table S3*.

As a sensitivity analysis, we considered incident HF during follow-up a competing event, following individuals until either first incident AF, HF, death, or end of follow-up. In this analysis, we observed 505 (2.0%) individuals with incident AF, 195 (0.7%) with incident HF, while 387 (1.4%) died prior to event or end of follow-up. Considering incident HF during follow-up, a competing event did not substantially alter our findings (see Supplementary data online, *Figure S7*). Results also remained consistent in sensitivity analysis excluding individuals with reduced LVEF (see Supplementary data online, *Table S2*).

### Associations with heart failure and stroke

During follow-up, 230 (0.9%) individuals were diagnosed with incident HF. Having one AtCM marker conferred a HR of 1.24 (95% CI: 0.89–1.74, *P* = .20) for HF, compared with referents with no markers. Individuals with ≥2 AtCM markers had a HR of 3.08 (95% CI: 2.03–4.66, *P* < .001) for HF compared with referents. Cumulative incidences are illustrated in *Figure 4B*. Censoring at incident AF attenuated the estimates. In sensitivity analyses having ≥2 AtCM markers remained associated with a higher HR for HF (HR: 2.55, 95% CI: 1.47–4.40, *P* < .001). Cumulative incidences with AF as a competing risk are illustrated in Supplementary data online, *Figure S8*.

Considering incident ischemic stroke as an outcome, we identified 171 (0.6%) individuals diagnosed with incident stroke during follow-up. We did not observe an association with stroke in individuals with only one AtCM marker (HR: 1.02, 95% CI: 0.66–1.58, *P* = .93), compared with referents. In individuals with ≥2 AtCM markers, we observed elevated rates of stroke (HR: 3.07, 95% CI: 1.78–5.28, *P* < .001). Cumulative incidences are visualized in *Figure 4C*. Attenuated estimates were observed when censoring at incident diagnosed AF, and only 154 incident strokes were observed. Here, individuals with ≥2 AtCM markers had a HR of 2.29 (95% CI: 1.19–4.39, *P* = .013) for incident ischemic stroke, compared with referents. Cumulative incidences considering AF as a competing risk are illustrated in Supplementary data online, *Figure S9*. Excluding individuals with reduced LVEF in a sensitivity analysis did not substantially alter the results for either HF or stroke (see Supplementary data online, *Table S2*). Hazard ratios for AF, HF, and stroke according to each AtCM marker evaluated separately are summarized in Supplementary data online, *Figure S10*.

### Model discrimination and calibration

The models incorporating AtCM markers exhibited *C*-statistics of 0.752 (95% CI: 0.710–0.790) for AF, 0.798 (95% CI: 0.735–0.848) for HF, and 0.676 (95% CI: 0.596–0.748) for stroke. For all outcomes, the inclusion of AtCM markers showed incremental improvements in model discrimination compared with reference models, with the largest changes observed for AF and HF. C-statistics are summarized in Supplementary data online, *Table S4*. Models appeared reasonably well-calibrated, although we noted a trend towards overestimating risk in individuals at a higher predicted risk. Calibration plots are shown in Supplementary data online, *Figure S11*.

### Integration with clinical and genetic risk

**Figure 5:**
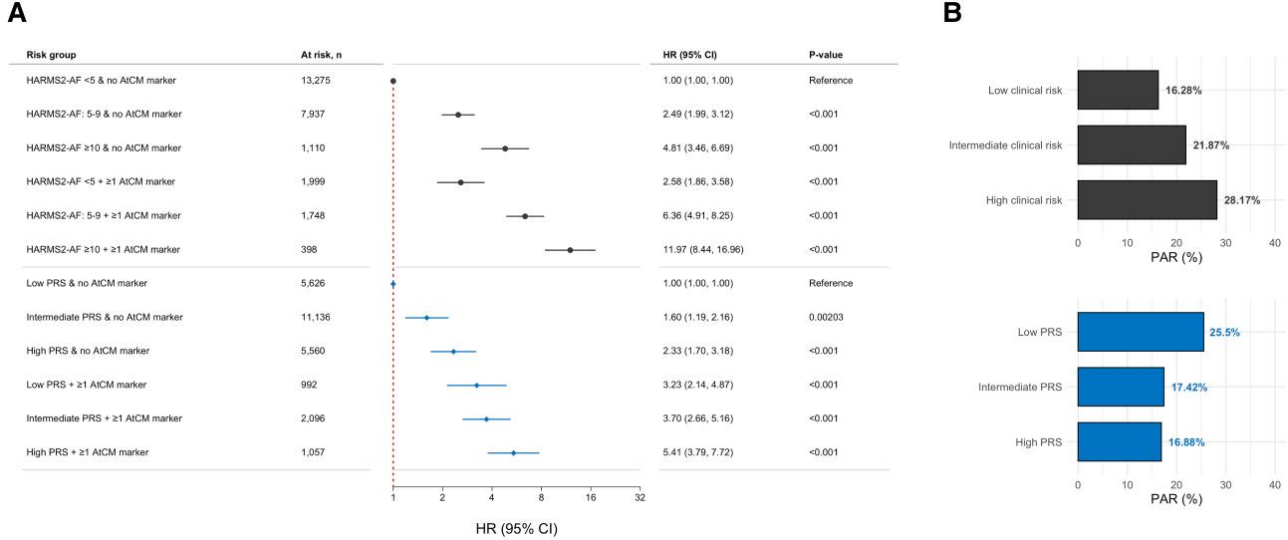
Integration with clinical and genetic risk. Panel *A* shows hazard ratios and confidence intervals for atrial fibrillation, stratified by the presence of ≥1 AtCM marker, and clinical risk and genetic risk scores. Dotted line represents a hazard ratio of 1.0. Clinical risk was stratified by the HARMS2-AF score. Panel *B* shows the population attributable risk (PAR) of having ≥1 AtCM marker, stratified by clinical and genetic risk. AtCM, atrial cardiomyopathy; CI, confidence interval; HR, hazard ratio; PRS, polygenic risk score

Stratified by the HARMS2-AF clinical risk score, higher rates of AF were observed in individuals with high a clinical risk (HR: 4.88, 95% CI: 3.57–6.66, *P* < .001), compared with referents with low clinical risk score (*Figure 5*). Presence of an AtCM marker was associated with an additional risk of AF. Individuals with both high clinical risk and an AtCM marker had a HR of 14.65 for AF (95% CI: 10.23–20.97, *P* < .001), compared with referents with low clinical risk and no markers of AtCM.

A similar trend was observed when integrating AtCM markers with a PRS representing genetic risk of AF. Individuals with a high PRS had a HR of 2.37 for AF (95% CI: 1.76–3.18, *P* < .001), compared with those with a low PRS. Among those with both high PRS and an AtCM marker, an increased HR for AF was observed (HR: 6.87, 95% CI: 4.79–9.85, *P* < .001) when compared with referents with low PRS and no markers of AtCM. Estimates for all clinical and genetic risk strata are summarized in *Figure 5A*. Cumulative incidences across clinical and genetic risk strata are visualized in Supplementary data online, *Figs S12* and *S13*. Estimates from analyses with further stratification for number of AtCM markers revealed a risk gradient according to increasing number of AtCM markers across both clinical and genetic risk strata (see Supplementary data online, *Tables S5* and *S6* and Supplementary data online, *Figure S14*).

For incident stroke and HF, the presence of ≥1 AtCM marker was associated with increased rates of both outcomes among individuals at intermediate and high genetic risk. No significant associations were observed among individuals at low genetic risk. HRs for both outcomes stratified by genetic risk and presence of ≥1 AtCM marker are summarized in Supplementary data online, *Figure S15*.

### Reclassification of atrial fibrillation according to atrial cardiomyopathy markers

The addition of AtCM markers resulted in an NRI of 13.7% (95% CI: 9.2%–18.3%) for incident AF, compared with a reference model adjusted for sex, age, BMI, CAD, hypertension, and diabetes. When compared to a model adjusted for the HARMS2-AF clinical risk score, further stratification by the presence of ≥1 AtCM marker resulted in an NRI of 8.1% (95% CI: 3.5%–12.6%). Compared with genetic risk of AF, considering the presence of ≥1 AtCM marker, yielded an NRI of 7.3% (95% CI: 4.6%–10.2%).

### Combined risk model for atrial fibrillation

**Figure 6:**
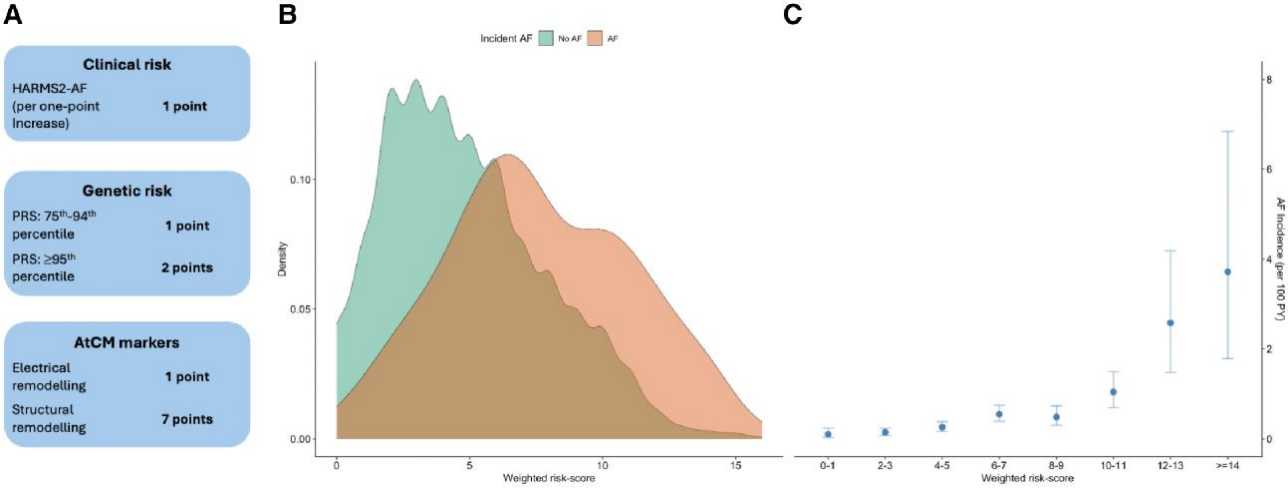
Integrated risk score for atrial fibrillation. Panel *A* shows the weighting of clinical and genetic risk factors together with markers of AtCM in the weighted risk score. Weights were determined in a 70% subset of the cohort. Clinical risk is per one-point increase in the HARMS2-AF score. Electrical remodelling comprises either prolonged P-wave duration or abnormal P-wave terminal force. Structural remodelling comprises either left atrial dilation or left atrial mechanical dysfunction. Panel *B* shows a density plot stratified by incident AF vs no incident AF for the remaining 30% of the cohort. Panel *C* shows AF incidence per 100 person-years according to two-point increment increases in the weighted risk score. AF, atrial fibrillation; AtCM, atrial cardiomyopathy; PRS, polygenic risk score; PY, person-years

In an exploratory analysis, we constructed a weighted risk score integrating both clinical and genetic risk with AtCM markers (*Figure 6A*). In a 30% subset of the cohort for testing, a one-point increase in this risk score was associated with a HR of 1.29 for AF (95% CI: 1.23–1.35, *P* < .001). A density plot of scores stratified by incident AF during follow-up vs no AF is shown in *Figure 6B*. We observed increasing incidence of AF at higher risk scores, as illustrated in *Figure 6C*. Internal validation using 200 bootstrap replicates showed yielded an optimism corrected *C*-statistic of 0.72 and an optimism calibration curve slope of 1.012 (optimism: −0.012).

### Population attributable risk of atrial fibrillation according to atrial cardiomyopathy markers

When stratifying the cohort by clinical risk, we observed a higher PAR for having ≥1 AtCM marker in strata with intermediate (21.87%) and high (28.17%) clinical risk (*Figure 5B*). An inverse trend was observed when stratifying by genetic risk instead; the highest PAR for AtCM was observed in the strata with a low PRS for AF (25.50%).

## Discussion

In the present study, we leveraged clinical information, ECGs, and cardiac imaging in more than 26 000 individuals, to ascertain markers of AtCM. We explored the prevalence and inter-relations of these markers and suggested threshold values for cardiac magnetic resonance-derived markers of AtCM. We examined risk factors for AtCM and found a dose-response-like relationship between the number of AtCM markers and incident AF, stroke, and HF. Importantly, the association with AF remained robust in sensitivity analyses considering HF as a competing risk. By integrating clinical risk scores and genomic data, we assessed the contribution of AtCM markers to AF risk and their PAR across cohort strata (*Structured Graphical Abstract*).

### Atrial cardiomyopathy as a common substrate for atrial fibrillation, stroke, and heart failure

In recent years, AtCM has increasingly been recognized as a distinct clinical entity and an important substrate for AF and stroke.^1,4^ Research has associated low voltage areas in the left atrium with a history of stroke in AF patients.^33^ Genetic evidence has also linked genetic variants in hallmark genes of cardiomyopathies with AF,^25^ and identified associations between the genetic underpinnings of LA structure and function with risk of both AF^34,35^ and stroke.^36^ Nonetheless, the relationship between AtCM and stroke risk is still not completely clear. One study in AF-free patients undergoing continuous cardiac monitoring found that changes in LA measures were associated with more white matter lesions and lacunar infarcts,^37^ hinting at an independent role of AtCM in cerebrovascular disease. In contrast, a recent study did not find any significant differences in LA measures in patients with embolic stroke of undetermined source (ESUS) compared with controls.^38^ Similarly, the recent ARCADIA trial found no significant effect of anticoagulation to prevent stroke recurrence in AF-free ESUS patients and evidence of AtCM.^39^

The results of the present study support a role of AtCM in both stroke and HF, with consistent findings in sensitivity analyses considering AF as a competing risk. This indicates that AtCM may be a substrate for stroke regardless of adjustment for interim AF in individuals with ≥2 AtCM markers, which is consistent with findings from clinical and genetic studies.^4,36^ Similar findings were observed for risk of HF, again with robust results in sensitivity analyses.

Our findings indicate that AtCM may be one of the final common pathways of AF, HF, and stroke. Notably only individuals with two or more markers of AtCM showed increased rates of HF and stroke, suggesting that preventive efforts may be most beneficial in individuals with several markers of AtCM.

### Atrial cardiomyopathy risk factors and sex differences

Male sex was associated with having at least one AtCM marker, and age at or above 65 years, and history of CAD and hypertension were all associated with AtCM. A high alcohol intake, a risk factor for AF,^21,40^ was also associated with increased OR of AtCM. The association between alcohol intake and AtCM seemed to be largely driven by LA dilation and P-wave prolongation, while other risk factors such as CAD and age were to a higher degree associated with reduced LA_EF_. Interestingly, a high alcohol intake was only associated with a prolonged P-wave duration in men, while women with a high alcohol intake showed a higher degree of LA dilation. We also noted a sex-specific difference for the association between obesity and AtCM, with opposite directions of effect in men and women. This could in part be due to differences in adipose tissue distribution between men and women, as gluteo-femoral fat, which is more abundant in women, may be less associated with cardiovascular disease risk^41–43^.

The risk factors identified in this study—age, hypertension, CAD, and alcohol intake—are well-established AF risk factors.^44^ Through adverse atrial remodelling (i.e. AtCM), these risk factors may set the stage for later development of AF, HF, and stroke. In general, there is an increased focus on reducing risk factor burden for cardiovascular disease, including AF.^21,45^ Consequently, addressing these risk factors could potentially slow or prevent the development and progression of AtCM, and its downstream clinical consequences. Prevention and risk factor modification would likely need to include an approach comprising both medical and non-medical interventions.^8,9^ As arrhythmia itself may also accelerate remodelling, catheter ablation could potentially also be relevant in halting or reversing AtCM progression, which is currently being investigated in an ongoing clinical trial (RACE X, ClinicalTrials.gov identifier: NCT06200311).^46^

### Clinical implications of atrial cardiomyopathy markers

Our observed reference ranges for CMR-derived LAVI_Max_ and LA_EF_ measures align well with previously reported measures from smaller cohorts,^19^ the UK Biobank,^47^ and a recent expert consensus paper from the European Association of Cardiovascular Imaging.^20^ As genetic determinants of LAVI_Min_ have previously been reported to be strongly associated with AF,^34^ we included both LAVI_Max_ > 60 mL/m^2^ and LAVI_Min_ > 30 mL/m^2^ as markers of LA dilation. Combined with reduced LA_EF_ as a marker of mechanical dysfunction, and P-wave prolongation, we revealed that almost 1 in 10 had at least one marker of AtCM, while overlap between markers was relatively modest.

P-wave prolongation and abnormal PTF were the most frequently observed AtCM markers but were associated with smaller increases in AF HRs compared to LA dilation and mechanical dysfunction, and were not robustly associated with HF or stroke. This illustrates a trade-off between easily available but more unspecific ECG markers, and imaging-based markers, which seem more specific, but are more expensive and unlikely to be as readily available.

When assessing the PAR of AtCM on AF risk, we observed the highest PAR in individuals that were also at high clinical risk of AF, indicating that this population group may benefit the most from screening and preventive efforts of AtCM and subsequent arrhythmia. Inversely, AtCM had a lower PAR for AF in those at high genetic risk of AF, compared with those with a lower genetic risk of AF. This could potentially be due to individuals with a high genetic predisposition for AF developing disease at an earlier age. Thus, as this study only considered incident AF, individuals with high genetic risk may have developed AF prior to enrolment at higher rates and were consequently not considered here.

Given these findings, screening for AtCM should likely focus on specific high-risk patient groups. Identifying these individuals early may facilitate targeted preventive strategies, reducing their risk of developing AF, HF, and stroke. Using a weighted risk score integrating AtCM markers with clinical and genetic risk in an exploratory analysis, we found that an integrative approach of combining both clinical and genetic risk factors with markers of AtCM could potentially aid in risk stratification. However, this risk score would need to be validated in independent cohorts. Our study provides a foundation for future research into the clinical utility of AtCM markers, although further research in independent cohorts is necessary to establish standardized diagnostic thresholds. Prospective studies could evaluate whether interventions targeting modifiable risk factors can reverse AtCM and reduce AF risk.

### Limitations

The present findings should be interpreted with consideration for the potential study limitations. Our study also only included individuals of European ancestry. While this may limit bias due to differences in LA volumes, and ECG measures, it also limits the generalizability of our findings to other population groups that may have differences in LA measures.^48^ Moreover, the general make-up of the UK Biobank could introduce a healthy-volunteer bias, possibly affecting the AtCM rate within the cohort and limiting generalizability. Moreover, the UK Biobank did not contain measures of atrial fibrosis, atrial strain, or continuous cardiac monitoring, and we were therefore unable to ascertain other postulated AtCM markers derived from these measures. Our measures of LA volumes were indexed according to BSA. While BSA-indexing of LA volumes has been recommended by cardiac imaging societies guidelines,^16^ we acknowledge that it may also lead to an underestimation of LA dilation severity in obese individuals.^49^ While the use of cMRI may have reduced the risk of operator bias compared with echocardiographic imaging, it cannot be assumed that the findings are directly translatable to echocardiographic imaging in a clinical setting. We used a complete-case approach and excluded individuals with missing measures. This could reduce generalizability, and if missingness of values was not random, but instead related to cardiovascular health and comorbidities, removing these individuals could potentially introduce bias. Currently the UK Biobank only contains serial imaging on a small subset of participants, which limited our ability to assess potential progression in AtCM. Similarly, only a small number of participants with imaging/ECG had simultaneous measures of plasma proteins. This limited our ability to integrate potential proteomic markers of AtCM, such as N-terminal pro-B-type natriuretic peptide. The analyses on PAR for AtCM also assume that AtCM is preventable or reversible, which warrants further study. Thus, the PAR analyses should be regarded as exploratory and hypothesis generating findings. The integrated risk score adopted was not externally validated and should therefore be regarded as an exploratory analysis. We acknowledge that we examined incident diagnosed AF; AF may be clinically unrecognized and undiagnosed. Hence, our competing risk analyses may not have accounted for undiagnosed AF. Finally, bias due to residual confounding cannot be ruled out.

## Conclusions

Imaging and ECG markers of AtCM were present in 15.7% of a large cohort without prior AF, HF, or stroke. Several well-established AF risk factors were associated with these AtCM markers. Atrial cardiomyopathy markers were associated with increased rates of AF, stroke, and HF, with higher rates in individuals with two or more markers. Integration of these markers with clinical and genetic risk scores showed an additional risk and identified the highest population attributable risk of AtCM in individuals with high clinical risk of AF. These findings support that AtCM is an important common substrate for the development of AF, HF, and stroke later in life, and support the notion that focusing on AtCM diagnosis and treatment may ultimately prevent AF, HF, and stroke.

## Supplementary Material

ehaf793_Supplementary_Data

## Declarations

### Data Availability

All data underlying this study are available through the UK Biobank. Bona-fide researchers can apply for access to the UK Biobank through https://www.ukbiobank.ac.uk/enable-your-research/apply-for-access.
