# Supplementary material for: Atrial cardiomyopathy: markers and outcomes
Source: Eur Heart J. 2025 Oct 15;47(2):235–46. doi: 10.1093/eurheartj/ehaf793 (PMC12777705; doi:10.1093/eurheartj/ehaf793)
Supplement: ehaf793_Supplementary_Data [file ehaf793_supplementary_data.zip › AtCM_suppl_2ndRev.pdf]

Supplemental material

Vad et al. Atrial cardiomyopathy: markers and outcomes

Index

Supplemental table 1. Data fields and phenotype definitions 2

Supplemental table 2. Rates of AF, HF, and Stroke, in individuals with LVEF >50% 3

Supplemental table 3. Rates of atrial fibrillation according to combinations of AtCM markers 3

Supplemental table 4. C-statistics for models including AtCM markers 4

Supplemental table 5. Rates of AF according to number of AtCM markers and clinical risk 5

Supplemental table 6. Rates of AF according to number of AtCM markers and genetic risk 5

Supplemental Figure 1. Flowchart of participant selection 6

Supplemental Figure 2. Influence of risk factors on AtCM markers 7

Supplemental Figure 3. Influence of risk factors on prolonged P-wave duration stratified by sex 8

Supplemental Figure 4. Influence of risk factors on abnormal P-wave terminal force stratified by sex 9

Supplemental Figure 5. Influence of risk factors on left atrial dilation stratified by sex 10

Supplemental Figure 6. Influence of risk factors on left atrial mechanical dysfunction stratified by sex 11

Supplemental Figure 7. Rates of incident AF with heart failure as competing event 12

Supplemental Figure 8. Rates of incident HF with AF as competing event 13

Supplemental Figure 9. Rates of incident ischemic stroke with AF as competing event 14

Supplemental Figure 10. Outcomes according to each atrial cardiomyopathy marker 15

Supplemental Figure 12. Incidence of AF according to AtCM markers and clinical risk 17

Supplemental Figure 13. Incidence of AF according to AtCM markers and genetic risk 18

Supplemental Figure 14. Rates of AF according to AtCM markers, and clinical and genetic risk 19

Supplemental Figure 15. Rates of HF and stroke according to genetic risk and AtCM markers 20

Supplemental table 1. Data fields and phenotype definitions

Phenotype/variable	UK Biobank data field(s)	Corresponding ICD10 code(s)
Sex	31, 22001	-
Age	21003	-
Ethnic background	21000	-
Genotype PCs	22009	-
Body-mass index	21001	-
Atrial fibrillation	131350	I48
Diabetes	130706, 130708, 130714	E10, E11, E14
Coronary artery disease	131296, 131298, 131300, 131304, 131306	I20, I21, I22, I24, I25
Chronic kidney disease	132033	N18
Chronic obstructive pulmonary disease	131491	J43
Heart failure	131354	I50
Hypertension	131286	I10
Ischemic stroke	131366, 131368	I63, I64
Sleep apnea	41280	G47.3
Alcohol intake	1558, 1568, 1578, 1588, 1598, 1608, 4407, 4418, 4429, 4440, 4451	-
Smoking status	20116	-
Left atrial volumes	24110, 24111	-
Left atrial emptying fraction	24113	-
P-wave duration	12338	-
P-wave terminal force	20205	-

PCs, principal components

Supplemental table 2. Rates of AF, HF, and Stroke, in individuals with LVEF >50%

**Outcome**	**AtCM Markers**	**HR (95% CI)**	**P**
Atrial Fibrillation	None	Reference	–
Atrial Fibrillation	1 AtCM marker	1.68 (1.32–2.13)	<0.001
Atrial Fibrillation	≥2 AtCM markers	4.41 (3.22–6.04)	<0.001
Heart failure	None	Reference	–
Heart failure	1 AtCM marker	1.18 (0.75–1.85)	0.48423
Heart failure	≥2 AtCM markers	3.61 (2.11–6.19)	<0.001
Ischemic stroke	None	Reference	–
Ischemic stroke	1 AtCM marker	1.23 (0.77–1.95)	0.38612
Ischemic stroke	≥2 AtCM markers	2.86 (1.48–5.51)	0.00172

AtCM, atrial cardiomyopathy, CI, confidence interval, HR, hazard ratio.

Supplemental table 3. Rates of atrial fibrillation according to combinations of AtCM markers

AtCM markers	Number at risk	HR	95% CI	P
P-prolongation + abnormal PTF	220	2.10	[1.18; 3.73]	0.011
P-prolongation + LA dilation	90	3.74	[1.99; 7.01]	<0.001
P-prolongation + LA mechanical dysfunction	16	NA	NA	NA
Abnormal PTF + LA dilation	93	4.62	[2.29; 9.31]	<0.001
Abnormal PTF + LA mechanical dysfunction	17	6.78	[1.69; 27.23]	0.007
LA dilation + LA mechanical dysfunction	183	6.03	[4.23; 8.58]	<0.001

AtCM, atrial cardiomyopathy, CI, confidence interval, HR, hazard ratio, LA, left atria, PTF, P-wave terminal force

Supplemental table 4. C-statistics for models including AtCM markers

Outcome	Model	C-index (95% CI)	Δ C-index
Atrial Fibrillation	Sex+age+BMI	0.724 (0.681 - 0.764)	Reference
Atrial Fibrillation	Sex+age+BMI +HT+CAD+Diabetes	0.733 (0.690 - 0.771)	0.009
Atrial Fibrillation	Sex+age+BMI +HT+CAD+Diabetes +AtCM markers	0.752 (0.710 - 0.790)	0.028
Heart Failure	Sex+age+BMI	0.732 (0.666 - 0.790)	Reference
Heart Failure	Sex+age+BMI +HT+CAD+Diabetes	0.787 (0.723 - 0.839)	0.055
Heart Failure	Sex+age+BMI +HT+CAD+Diabetes +AtCM markers	0.798 (0.735 - 0.848)	0.066
Stroke	Sex+age+BMI	0.667 (0.587 - 0.739)	Reference
Stroke	Sex+age+BMI +HT+CAD+Diabetes	0.669 (0.589 - 0.741)	0.002
Stroke	Sex+age+BMI +HT+CAD+Diabetes +AtCM markers	0.676 (0.596 - 0.748)	0.009

AtCM, atrial cardiomyopathy, BMI, body-mass index, CAD, coronary artery disease, HT, hypertension.

Supplemental table 5. Rates of AF according to number of AtCM markers and clinical risk

Clinical risk	Number of AtCM markers	HR (95% CI)	P
Low	None	Reference	–
Intermediate	None	2.49 (1.99–3.12)	<0.001
High	None	4.81 (3.46–6.69)	<0.001
Low	1 AtCM marker	2.11 (1.46–3.05)	<0.001
Intermediate	1 AtCM marker	4.59 (3.40–6.21)	<0.001
High	1 AtCM marker	10.63 (7.10–15.91)	<0.001
Low	≥2 AtCM markers	6.13 (3.53–10.64)	<0.001
Intermediate	≥2 AtCM markers	16.23 (11.38–23.16)	<0.001
High	≥2 AtCM markers	16.63 (9.40–29.43)	<0.001

AtCM, atrial cardiomyopathy, CI, confidence interval, HR, hazard ratio

Supplemental table 6. Rates of AF according to number of AtCM markers and genetic risk

Genetic risk	Number of AtCM markers	HR (95% CI)	p-value
Low	None	Ref	–
Intermediate	None	1.60 (1.19–2.16)	0.00204
High	None	2.33 (1.70–3.18)	<0.001
Low	1 AtCM marker	2.65 (1.67–4.22)	<0.001
Intermediate	1 AtCM marker	2.93 (2.04–4.21)	<0.001
High	1 AtCM marker	4.27 (2.86–6.38)	<0.001
Low	≥2 AtCM markers	6.24 (3.34–11.66)	<0.001
Intermediate	≥2 AtCM markers	8.09 (5.19–12.61)	<0.001
High	≥2 AtCM markers	9.75 (6.08–15.63)	<0.001

AtCM, atrial cardiomyopathy, CI, confidence interval, HR, hazard ratio

Supplemental Figure 1. Flowchart of participant selection

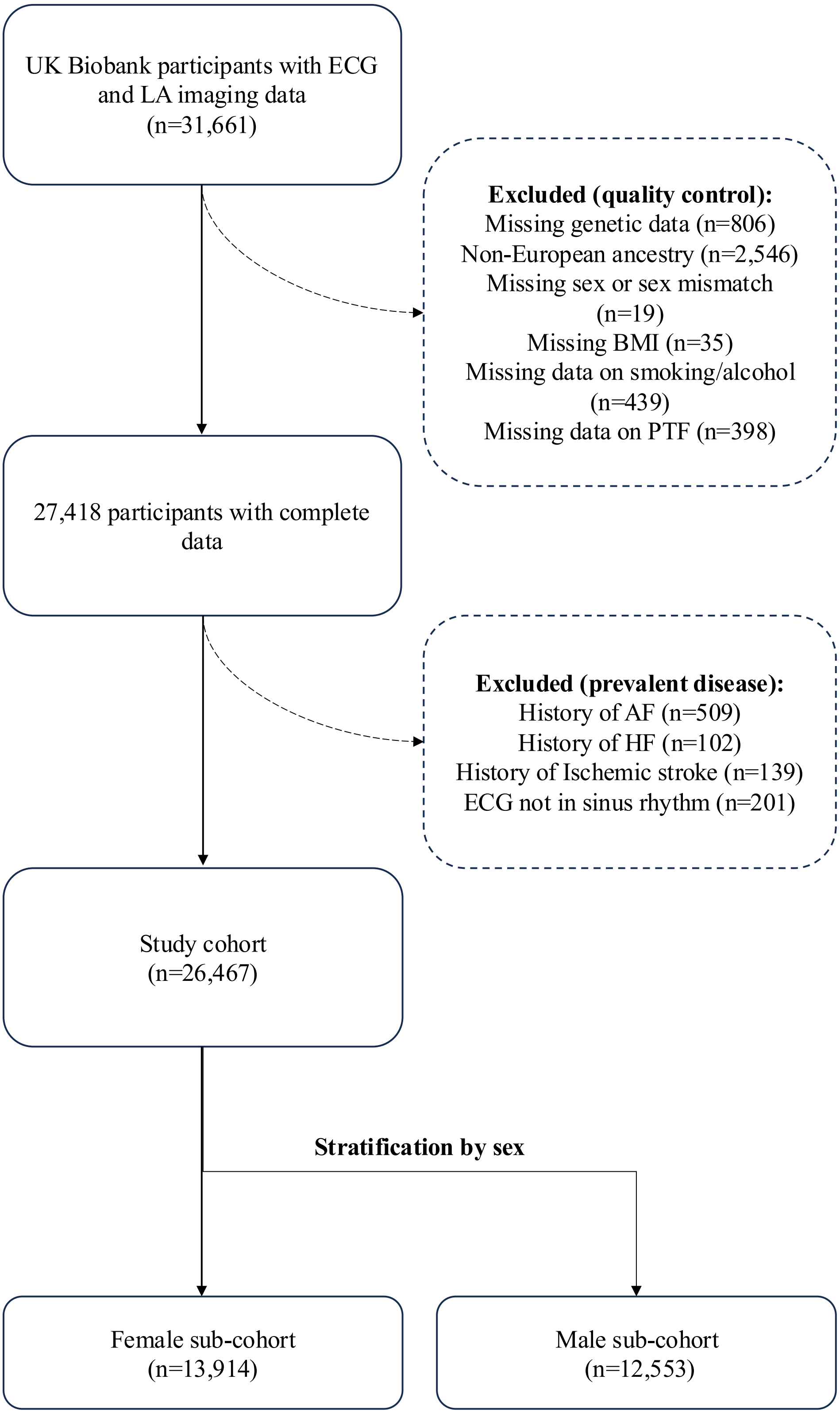

AF, atrial fibrillation, BMI, body-mass index, HF, heart failure, LA, left atria, PTF, P-wave terminal force

Supplemental Figure 2. Influence of risk factors on AtCM markers

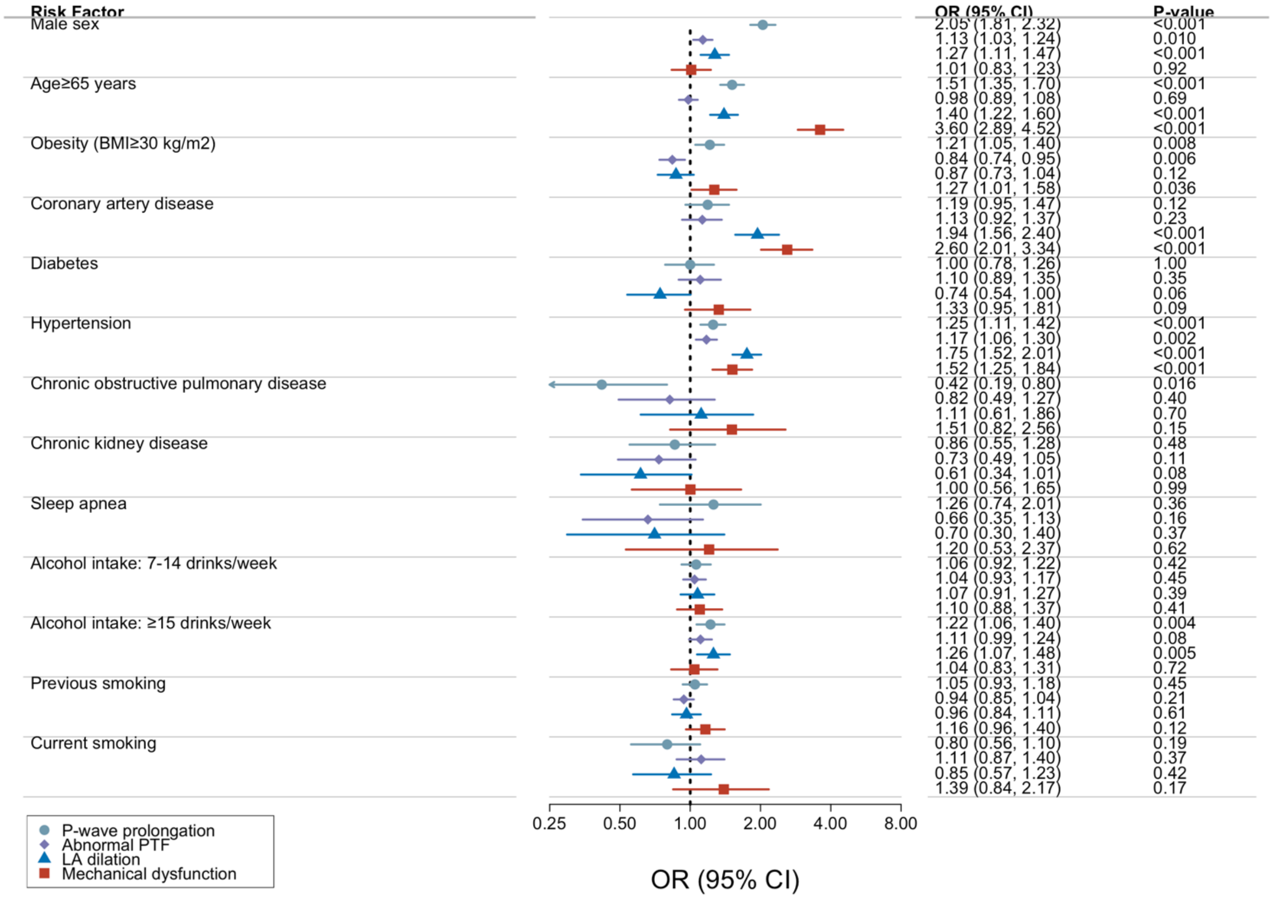

Associations between AF risk factors and individual AtCM markers. Associations with P-wave prolongation are shown with grey circles. Associations with abnormal P-wave terminal force are shown with purple diamonds. Associations with left atrial dilation are shown with blue triangles. Associations with reduced LA emptying fraction (mechanical dysfunction), are denoted with red squares. CI, confidence interval, LA, left atria, OR, odds ratio, PTF, P-wave terminal force.

Supplemental Figure 3. Influence of risk factors on prolonged P-wave duration stratified by sex

Risk Factor	OR (95% CI)	P-value	P-interaction
Age≥65 years	1.49 (1.23, 1.81)	<0.001	0.59
	1.52 (1.31, 1.76)	<0.001	
Obesity (BMI≥30 kg/m2)	0.96 (0.74, 1.24)	0.78	0.085
	1.34 (1.13, 1.59)	<0.001	
Coronary artery disease	1.57 (0.98, 2.40)	0.046	0.11
	1.12 (0.87, 1.42)	0.38	
Diabetes	0.97 (0.55, 1.58)	0.90	0.99
	1.02 (0.77, 1.33)	0.89	
Hypertension	1.42 (1.14, 1.77)	0.001	0.19
	1.18 (1.02, 1.37)	0.030	
Chronic obstructive pulmonary disease	0.22 (0.01, 0.98)	0.13	0.49
	0.49 (0.21, 0.97)	0.07	
Chronic kidney disease	0.89 (0.42, 1.66)	0.74	0.74
	0.85 (0.48, 1.40)	0.55	
Sleep apnea	2.15 (0.51, 6.03)	0.21	0.38
	1.14 (0.64, 1.89)	0.64	
Alcohol intake: 7-14 drinks/week	1.03 (0.82, 1.28)	0.82	0.81
	1.09 (0.90, 1.32)	0.36	
Alcohol intake: ≥15 drinks/week	0.95 (0.72, 1.23)	0.70	0.049
	1.34 (1.14, 1.59)	<0.001	
Previous smoking	1.20 (0.97, 1.46)	0.09	0.27
	0.99 (0.85, 1.14)	0.88	
Current smoking	0.54 (0.21, 1.12)	0.14	0.24
	0.87 (0.58, 1.25)	0.46	

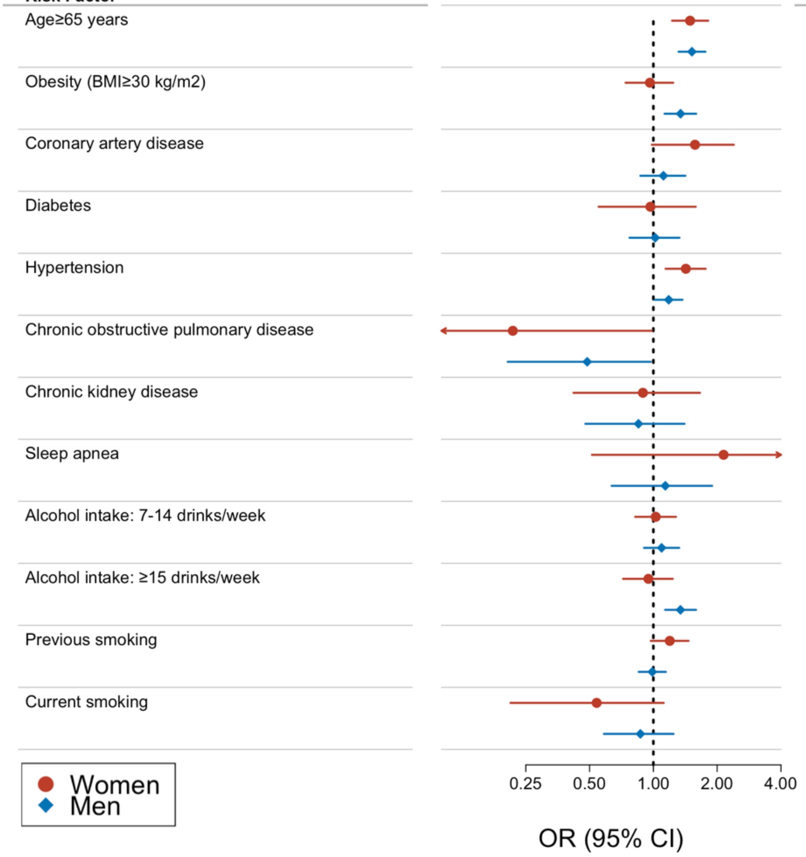

Associations between AF risk factors and prolonged P-wave duration. Multivariable models stratified by sex. Red denotes estimates for women, and blue denotes estimates for men. Black dotted line represents a reference odds ratio of 1.0. P-values for interaction with sex are shown in right-most column. CI, confidence intervals, OR, odds ratio.

Supplemental Figure 4. Influence of risk factors on abnormal P-wave terminal force stratified by sex

Risk Factor	OR (95% CI)	P-value	P-interaction
Age≥65 years	1.04 (0.91, 1.18)	0.61	0.22
	0.94 (0.82, 1.07)	0.34	
Obesity (BMI≥30 kg/m2)	0.73 (0.60, 0.88)	0.001	0.072
	0.94 (0.79, 1.11)	0.48	
Coronary artery disease	1.13 (0.77, 1.60)	0.51	0.84
	1.13 (0.89, 1.42)	0.30	
Diabetes	1.19 (0.82, 1.68)	0.33	0.65
	1.06 (0.81, 1.36)	0.66	
Hypertension	1.20 (1.03, 1.40)	0.019	0.70
	1.15 (1.00, 1.32)	0.044	
Chronic obstructive pulmonary disease	1.09 (0.53, 2.00)	0.80	0.21
	0.64 (0.30, 1.20)	0.20	
Chronic kidney disease	0.65 (0.35, 1.11)	0.14	0.60
	0.82 (0.47, 1.34)	0.47	
Sleep apnea	1.36 (0.41, 3.41)	0.56	0.15
	0.52 (0.23, 0.99)	0.07	
Alcohol intake: 7-14 drinks/week	1.09 (0.94, 1.26)	0.26	0.32
	0.98 (0.83, 1.16)	0.84	
Alcohol intake: ≥15 drinks/week	1.14 (0.96, 1.35)	0.14	0.48
	1.07 (0.92, 1.24)	0.38	
Previous smoking	0.96 (0.83, 1.11)	0.62	0.47
	0.92 (0.80, 1.05)	0.21	
Current smoking	1.40 (0.99, 1.95)	0.050	0.068
	0.92 (0.65, 1.26)	0.61	

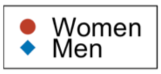

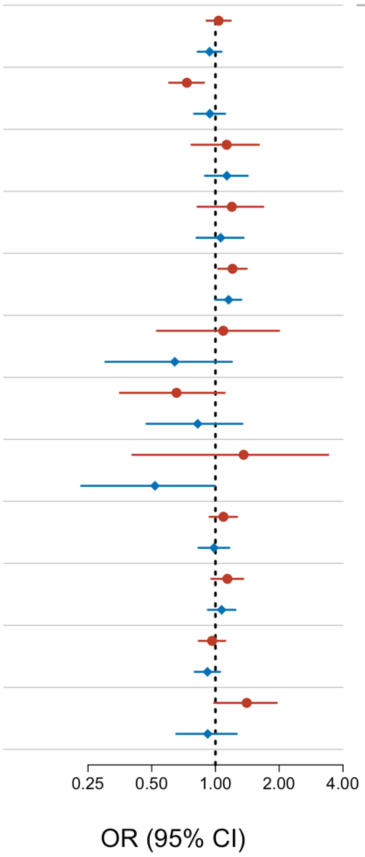

Associations between AF risk factors and abnormal P-wave terminal force. Multivariable models stratified by sex. Red denotes estimates for women, and blue denotes estimates for men. Black dotted line represents a reference odds ratio of 1.0. P-values for interaction with sex are shown in right-most column. CI, confidence intervals, OR, odds ratio

Supplemental Figure 5. Influence of risk factors on left atrial dilation stratified by sex

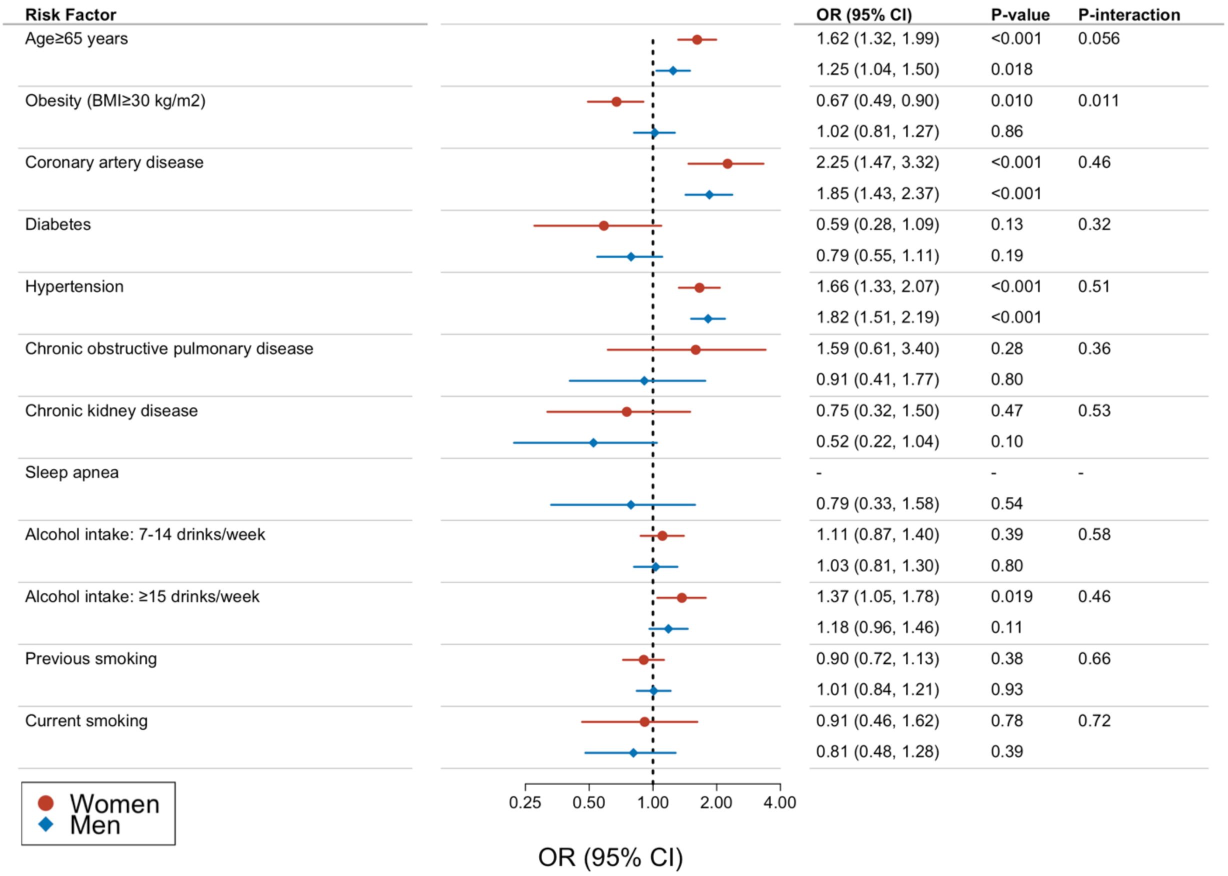

Associations between AF risk factors and left atrial dilation. Multivariable models stratified by sex. Red denotes estimates for women, and blue denotes estimates for men. Black dotted line represents a reference odds ratio of 1.0. Note that not enough women with sleep apnea had left atrial dilation for statistical comparison. P-values for interaction with sex are shown in right-most column. CI, confidence intervals, OR, odds ratio

Supplemental Figure 6. Influence of risk factors on left atrial mechanical dysfunction stratified by sex

Risk Factor	OR (95% CI)	P-value	P-interaction
Age≥65 years	3.59 (2.64, 4.95)	<0.001	0.96
	3.59 (2.63, 4.99)	<0.001	
Obesity (BMI≥30 kg/m2)	1.11 (0.78, 1.55)	0.55	0.42
	1.41 (1.05, 1.88)	0.021	
Coronary artery disease	2.75 (1.71, 4.24)	<0.001	0.73
	2.57 (1.88, 3.47)	<0.001	
Diabetes	1.17 (0.61, 2.05)	0.61	0.71
	1.40 (0.94, 2.02)	0.09	
Hypertension	1.73 (1.29, 2.30)	<0.001	0.28
	1.35 (1.04, 1.76)	0.026	
Chronic obstructive pulmonary disease	1.67 (0.57, 3.91)	0.29	0.86
	1.42 (0.65, 2.73)	0.33	
Chronic kidney disease	0.87 (0.34, 1.86)	0.75	0.76
	1.13 (0.52, 2.15)	0.73	
Sleep apnea	3.37 (0.78, 9.98)	0.05	0.090
	0.84 (0.29, 1.92)	0.71	
Alcohol intake: 7-14 drinks/week	1.07 (0.78, 1.45)	0.69	0.67
	1.16 (0.84, 1.59)	0.37	
Alcohol intake: ≥15 drinks/week	1.15 (0.79, 1.65)	0.45	0.77
	1.01 (0.75, 1.36)	0.94	
Previous smoking	0.96 (0.71, 1.28)	0.77	0.092
	1.36 (1.05, 1.76)	0.019	
Current smoking	1.20 (0.50, 2.45)	0.64	0.68
	1.55 (0.82, 2.70)	0.15	

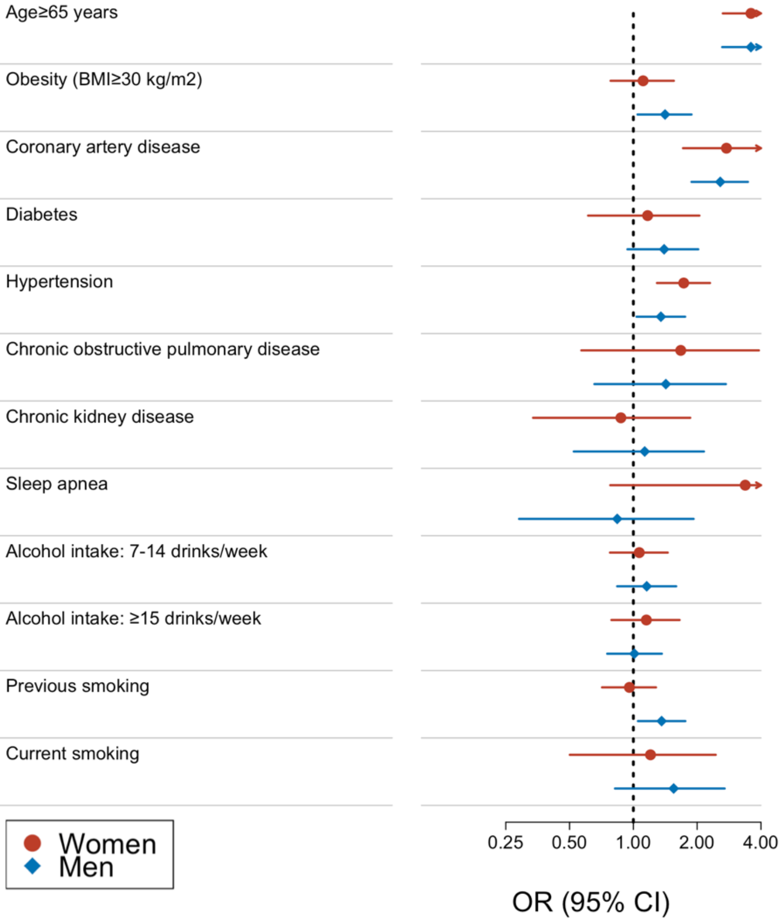

Associations between AF risk factors and abnormal P-wave terminal force. Multivariable models stratified by sex. Red denotes estimates for women, and blue denotes estimates for men. Black dotted line represents a reference odds ratio of 1.0. P-values for interaction with sex are shown in right-most column. CI, confidence intervals, OR, odds ratio

Supplemental Figure 7. Rates of incident AF with heart failure as competing event

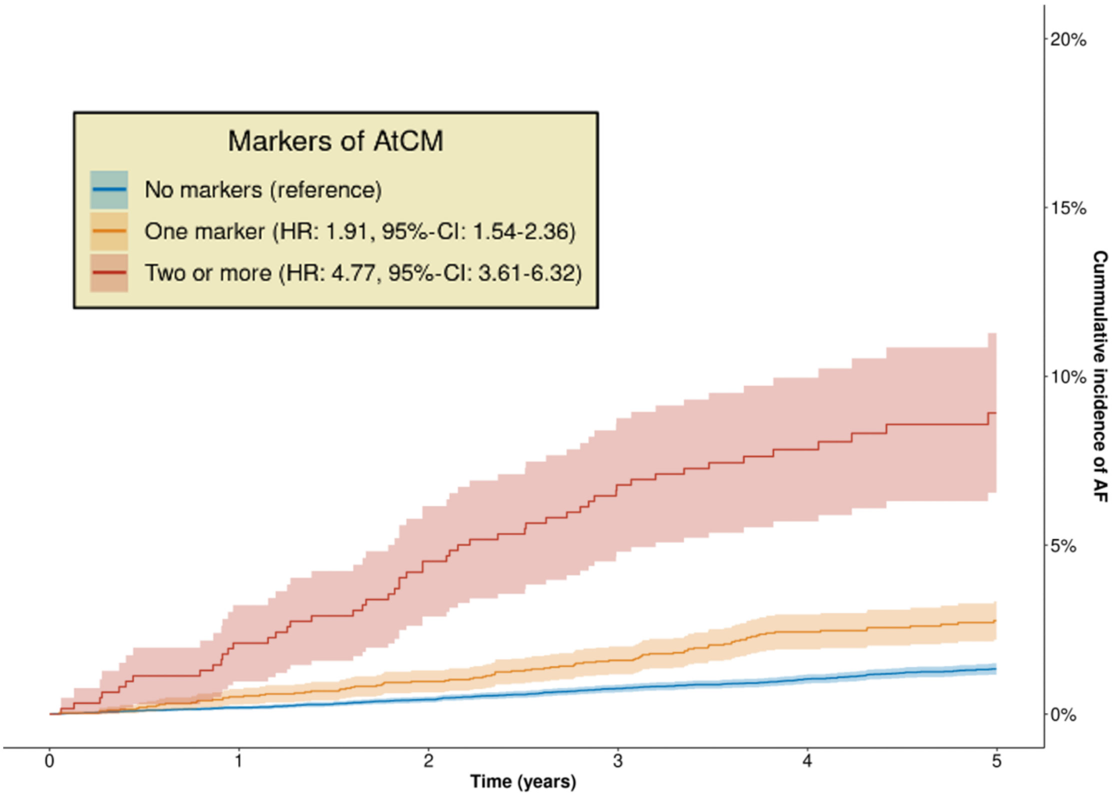

0 AtCM markers	22,322	22,239	22,122	21,964	16,239	10,850
1 AtCM marker	3,526	3,497	3,463	3,419	2,498	1,666
≥2 AtCM markers	619	598	577	558	391	250

Cumulative incidence and 95% confidence intervals for atrial fibrillation (AF) stratified by number of atrial cardiomyopathy markers. Analyses considered incident heart failure during follow-up as a competing risk. In this sensitivity analysis, higher rates of AF remained in individuals with one marker (orange), and in those with two or more markers (red), compared with referents (blue). Table at bottom show number at risk during follow-up. AtCM, atrial cardiomyopathy, CI, confidence interval, HR, hazard ratio

Supplemental Figure 8. Rates of incident HF with AF as competing event

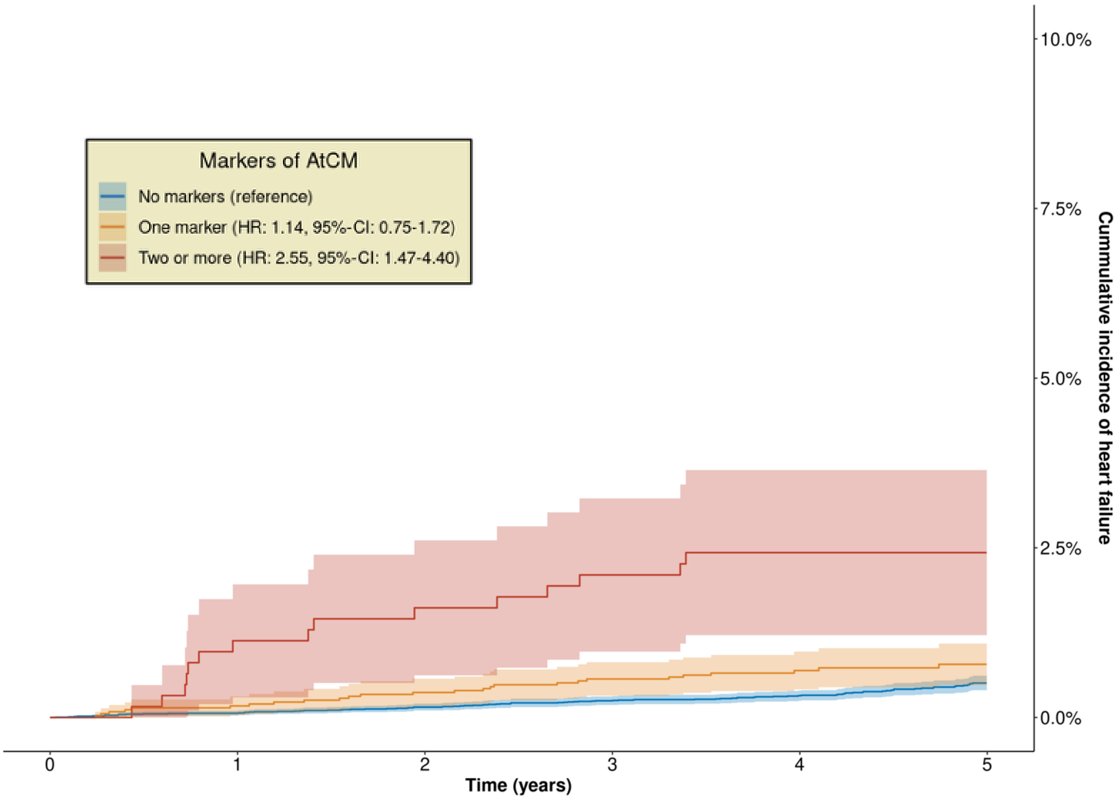

0 AtCM markers	22,322	22,239	22,122	21,964	16,239	10,850
1 AtCM marker	3,526	3,497	3,463	3,419	2,498	1,666
≥2 AtCM markers	619	598	577	558	391	250

Cumulative incidence for heart failure (HF) stratified by number of atrial cardiomyopathy markers. Analyses considered incident atrial fibrillation during follow-up as a competing risk. Individuals with one marker are shown in orange, and in those with two or more markers are shown in red. Individuals with no markers are shown in blue. Table at bottom show number at risk during follow-up. AtCM, atrial cardiomyopathy, CI, confidence interval, HR, hazard ratio

Supplemental Figure 9. Rates of incident ischemic stroke with AF as competing event

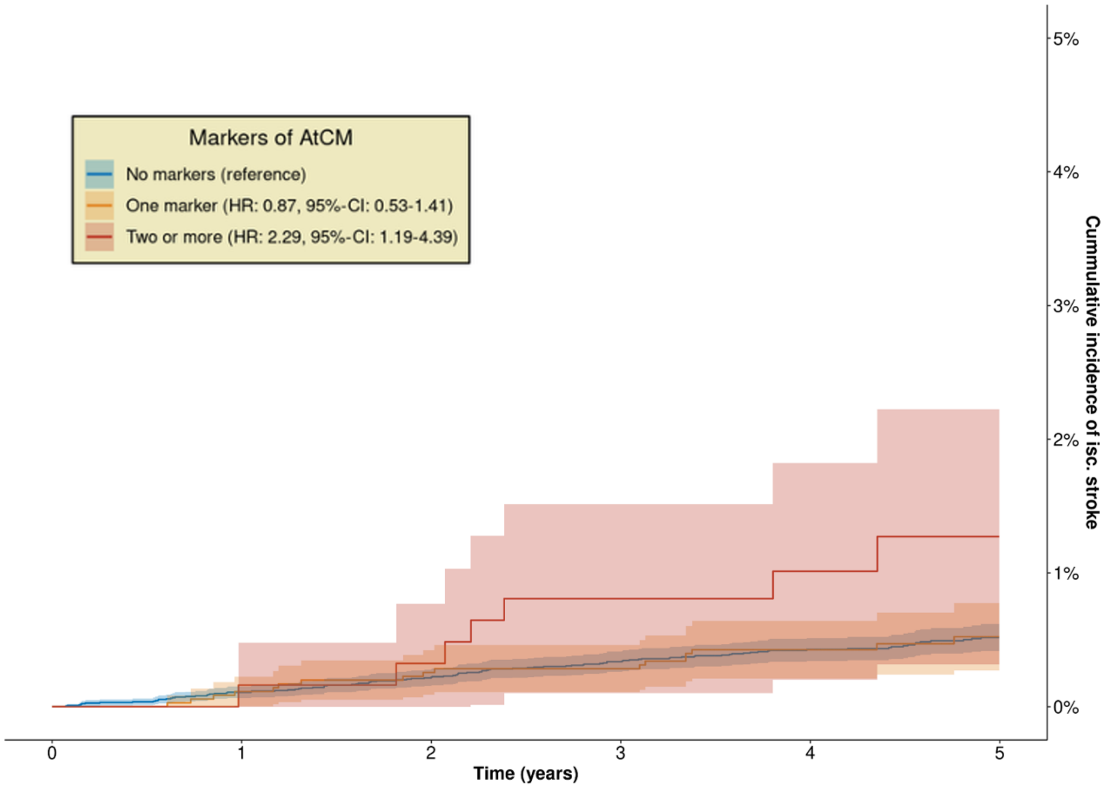

0 AtCM markers	22,322	22,228	22,111	21,944	16,225	10,853
1 AtCM marker	3,526	3,497	3,464	3,425	2,497	1,667
≥2 AtCM markers	619	603	583	563	396	252

Cumulative incidence for ischemic stroke stratified by number of atrial cardiomyopathy markers. Analyses considered incident atrial fibrillation during follow-up as a competing risk. Individuals with one marker are shown in orange, and in those with two or more markers are shown in red. Individuals with no markers are shown in blue. Table at bottom show number at risk during follow-up. AtCM, atrial cardiomyopathy, CI, confidence interval, HR, hazard ratio

Supplemental Figure 10. Outcomes according to each atrial cardiomyopathy marker

Marker	Outcome	HR (95% CI)	P-value
Prolonged P-wave	Atrial fibrillation	1.56 (1.17, 2.07)	0.002
Prolonged P-wave	Heart failure	0.64 (0.35, 1.17)	0.149
Prolonged P-wave	Ischemic stroke	1.09 (0.59, 2.01)	0.79
Abnormal PTF	Atrial fibrillation	1.42 (1.09, 1.84)	0.008
Abnormal PTF	Heart failure	1.38 (0.93, 2.05)	0.111
Abnormal PTF	Ischemic stroke	0.99 (0.57, 1.71)	0.96
LA dilation	Atrial fibrillation	4.00 (3.17, 5.04)	<0.001
LA dilation	Heart failure	3.07 (2.15, 4.40)	<0.001
LA dilation	Ischemic stroke	2.45 (1.47, 4.06)	<0.001
LA mechanical dysfunction	Atrial fibrillation	5.05 (3.91, 6.52)	<0.001
LA mechanical dysfunction	Heart failure	2.77 (1.81, 4.23)	<0.001
LA mechanical dysfunction	Ischemic stroke	3.54 (2.05, 6.10)	<0.001

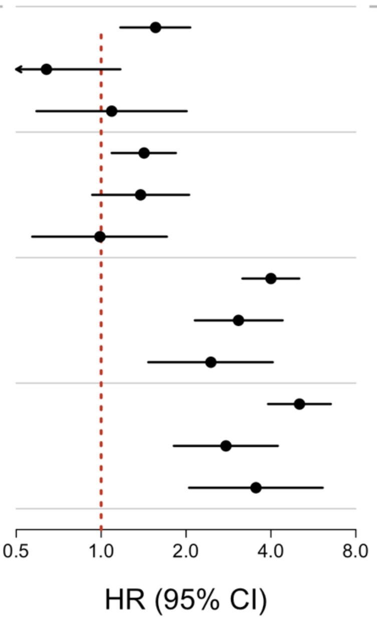

Hazard ratios for incident atrial fibrillation, heart failure, and stroke, according to each AtCM marker. AtCM, atrial cardiomyopathy, CI, confidence interval, HR, hazard ratio, LA, left atria, PTF, P-wave terminal force

Supplemental Figure 11. Incidence of AF according to AtCM markers and clinical risk

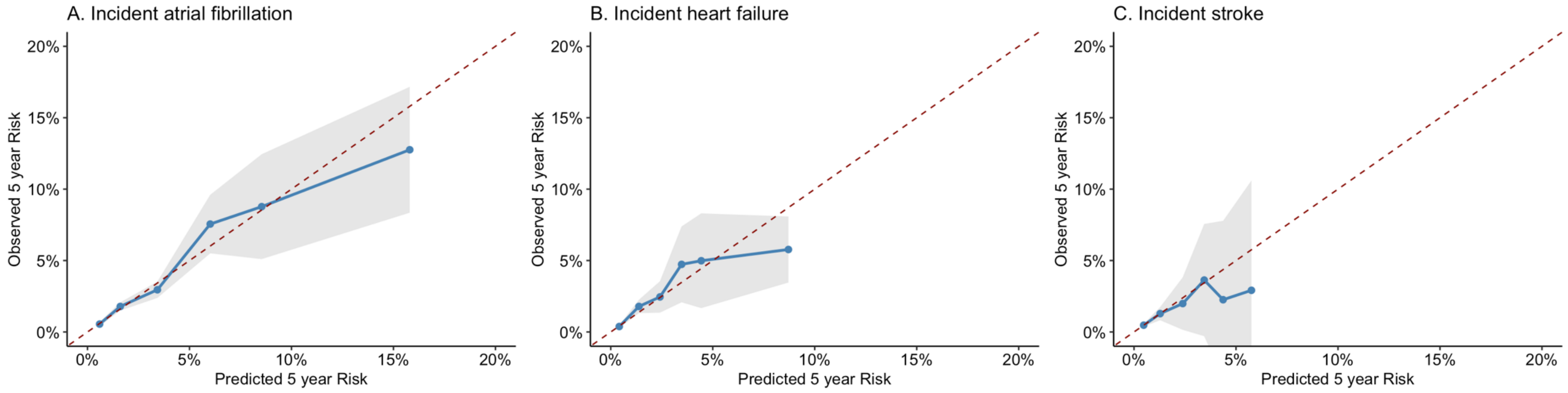

Panel A-C shows calibration curves for AF, HF, and stroke respectively. X-axis denotes predicted risk at 5 years of follow-up, while Y-axis denotes observed risk. Blue line represents the calibration curve, with the grey areas showing 95% confidence intervals. The red dotted lines shows the ideal curve.

Supplemental Figure 12. Incidence of AF according to AtCM markers and clinical risk

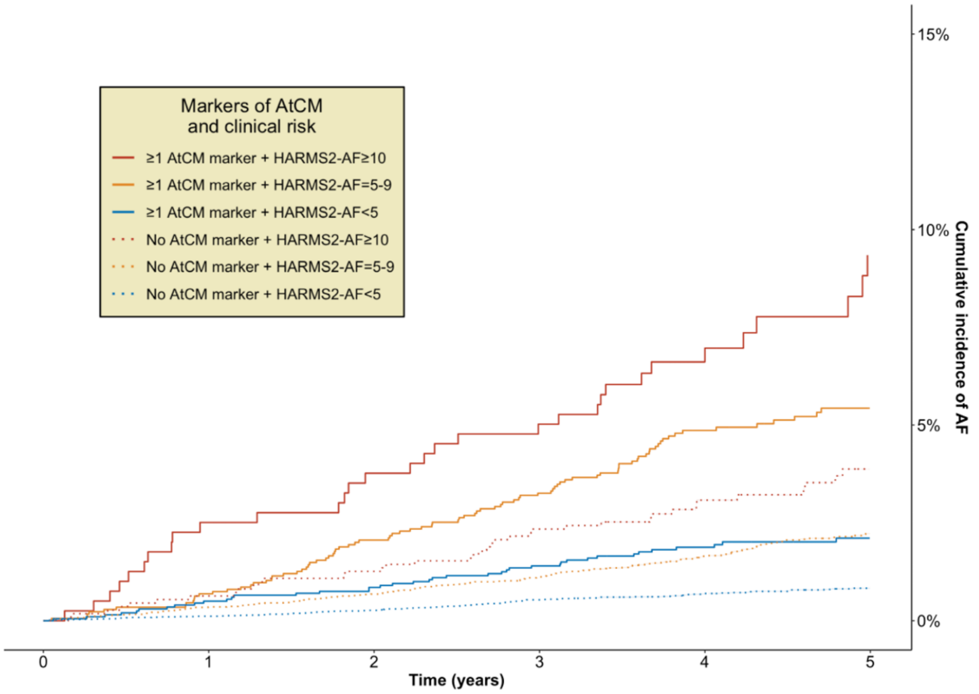

0 AtCM markers HARMS2-AF <5	13,275	13,249	13,212	13,151	9,785	6.648
≥1 AtCM markers HARMS2-AF <5	1,999	1,986	1,975	1,960	1,441	970
0 AtCM markers HARMS2-AF: 5-9	7,937	7,900	7,853	7,790	5,736	3,758
≥1 AtCM markers HARMS2-AF: 5-9	1,748	1,732	1,701	1,669	1,207	793
0 AtCM markers HARMS2-AF ≥10	1,110	1,101	1,087	1,067	765	491
≥1 AtCM markers HARMS2-AF ≥10	398	387	381	372	260	167

Cumulative incidence of AF stratified by ≥1 atrial cardiomyopathy markers (solid lines), and no atrial cardiomyopathy markers (dotted lines). Cohort was further stratified by $HARMS_2$-AF score ≥10 (red), $HARMS_2$-AF score between 5-9 (orange), and $HARMS_2$-AF score <5 (blue). Table at bottom show number at risk during follow-up. AtCM, atrial cardiomyopathy, HR, hazard ratio

Supplemental Figure 13. Incidence of AF according to AtCM markers and genetic risk

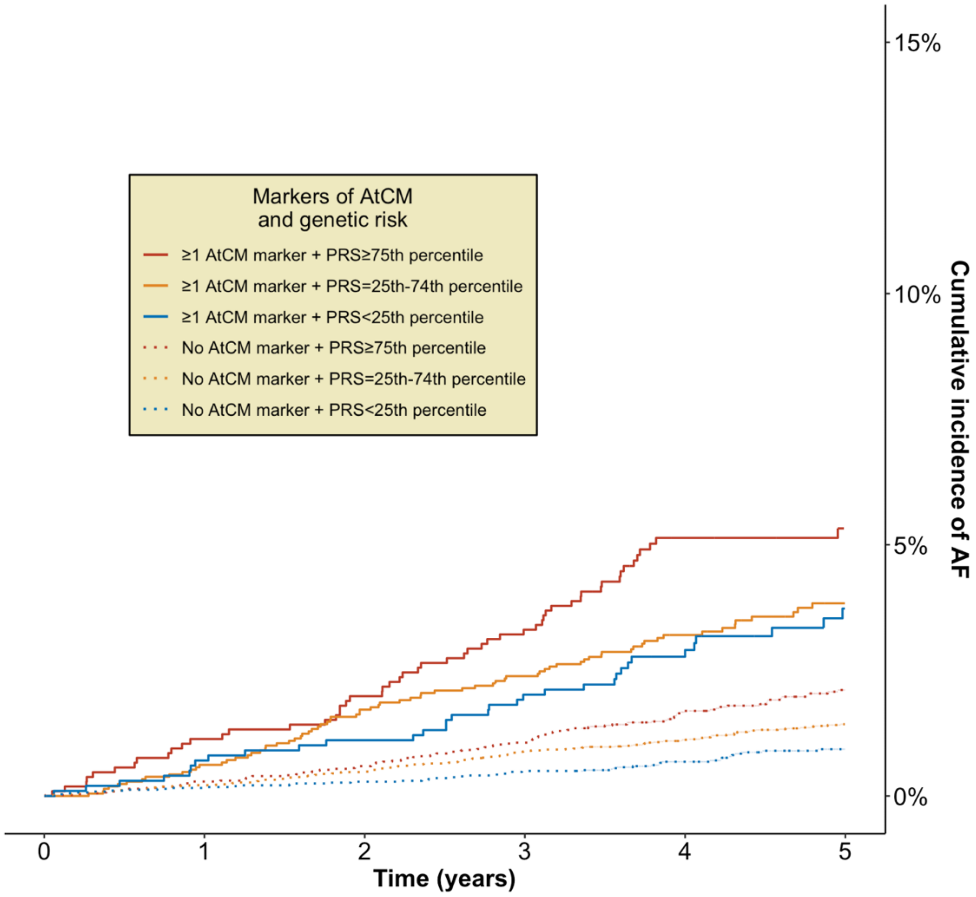

**0 AtCM markers PRS <25%**	**5,626**	**5,610**	**5,594**	**5,566**	**4,055**	**2,764**
**≥1 AtCM markers PRS <25%**	**992**	**981**	**973**	**962**	**714**	**482**
**0 AtCM markers PRS: 25-74%**	**11,136**	**11,103**	**11,051**	**10,978**	**8,184**	**5,449**
**≥1 AtCM markers PRS: 25-74%**	**2,096**	**2,081**	**2,051**	**2,026**	**1,466**	**964**
**0 AtCM markers PRS ≥75%**	**5,560**	**5,537**	**5,507**	**5,464**	**4,047**	**2,684**
**≥1 AtCM markers PRS ≥75%**	**1,057**	**1,043**	**1,033**	**1,013**	**728**	**484**

Cumulative incidence of AF stratified by ≥1 atrial cardiomyopathy markers (solid lines), and no atrial cardiomyopathy markers (dotted lines). Cohort was further stratified by PRS ≥75th percentile (red), PRS between 25th-74th percentile (orange), and PRS <25th percentile (blue). Table at bottom show number at risk during follow-up. AtCM, atrial cardiomyopathy, HR, hazard ratio.

Supplemental Figure 14. Rates of AF according to AtCM markers, and clinical and genetic risk

Clinical risk	Genetic risk	HR (95% CI)	P-value
Low	Low	1.00 (1.00, 1.00)	Reference
Low	Low	2.51 (1.21, 5.22)	0.013
Intermediate	Low	1.03 (0.59, 1.80)	0.91
Intermediate	Low	3.71 (2.05, 6.72)	<0.001
High	Low	1.32 (0.54, 3.26)	0.54
High	Low	5.21 (2.32, 11.71)	<0.001
Low	Intermediate	1.12 (0.70, 1.78)	0.65
Low	Intermediate	2.82 (1.61, 4.94)	<0.001
Intermediate	Intermediate	1.97 (1.26, 3.07)	0.003
Intermediate	Intermediate	4.16 (2.55, 6.80)	<0.001
High	Intermediate	3.06 (1.73, 5.39)	<0.001
High	Intermediate	6.32 (3.48, 11.50)	<0.001
Low	High	1.85 (1.15, 2.99)	0.011
Low	High	4.05 (2.20, 7.46)	<0.001
Intermediate	High	2.82 (1.77, 4.51)	<0.001
Intermediate	High	6.47 (3.86, 10.86)	<0.001
High	High	3.64 (1.94, 6.84)	<0.001
High	High	8.12 (4.09, 16.11)	<0.001

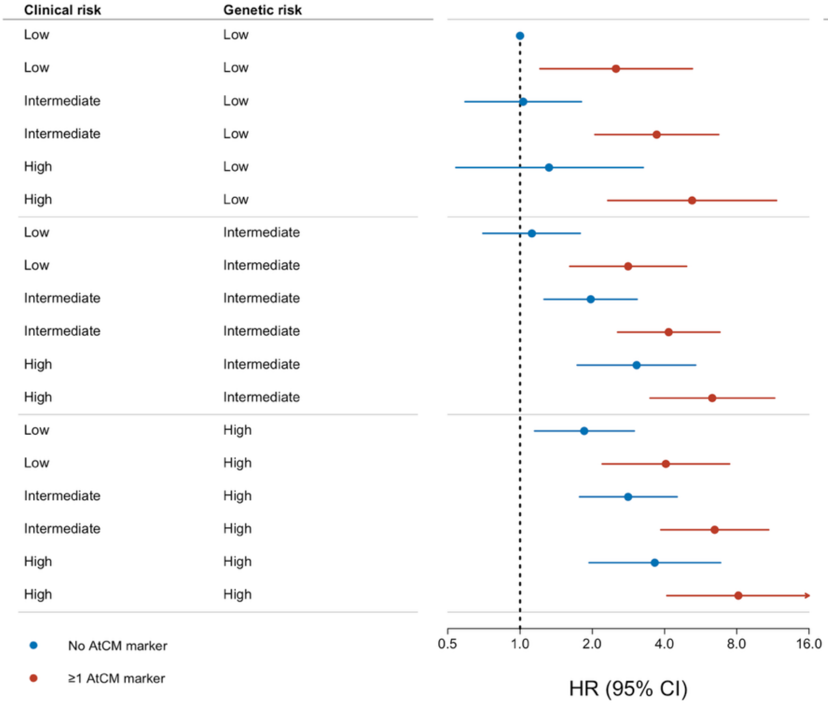

Hazard ratios for incident atrial fibrillation stratified by both clinical risk score, genetic risk, and presence of ≥1 AtCM marker. AtCM, atrial cardiomyopathy, CI, confidence interval, HR, hazard ratio

Supplemental Figure 15. Rates of HF and stroke according to genetic risk and AtCM markers

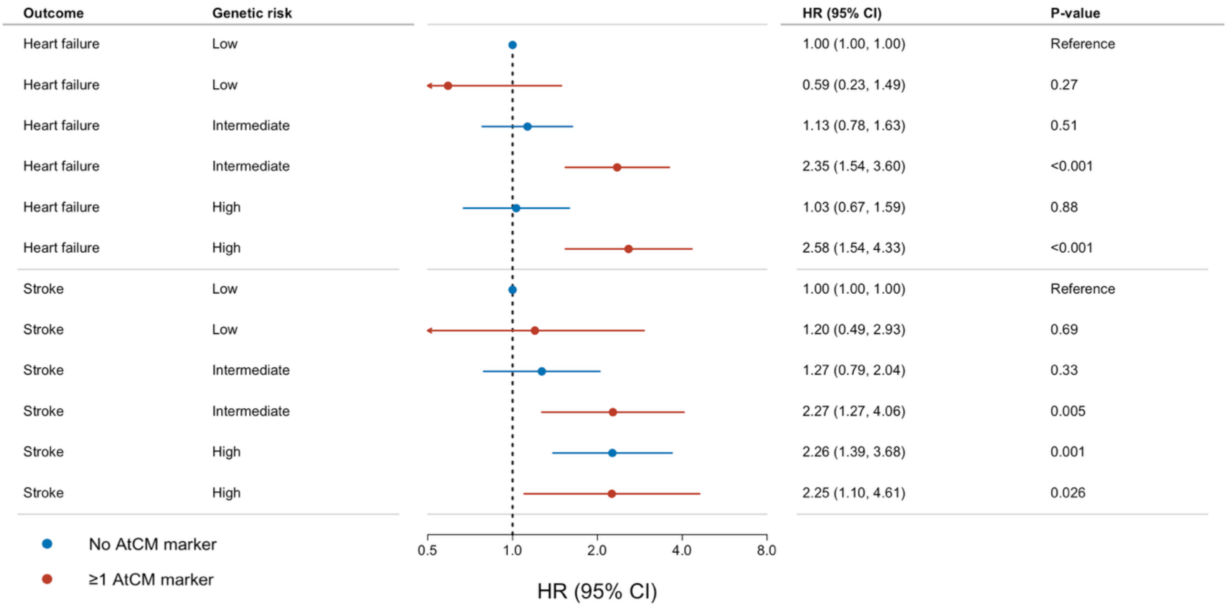

Hazard ratios for heart failure and stroke for individuals with ≥1 AtCM marker (red), compared with individuals with no AtCM markers (blue). The cohort was stratified by genetic predisposition for each respective outcome. Low genetic risk represents PRS <25th percentile. Intermediate genetic risk represents PRS between 25th and 74th percentile. High genetic risk represents PRS ≥75th percentile. AtCM, atrial cardiomyopathy, CI, confidence interval, HR, hazard ratio. PRS, polygenic risk score.
